# Participation of Acyl-Coenzyme A Synthetase FadD4 of *Pseudomonas aeruginosa* PAO1 in Acyclic Terpene/Fatty Acid Assimilation and Virulence by Lipid A Modification

**DOI:** 10.3389/fmicb.2021.785112

**Published:** 2021-11-16

**Authors:** Lorena Martínez-Alcantar, Gabriela Orozco, Alma Laura Díaz-Pérez, Javier Villegas, Homero Reyes-De la Cruz, Ernesto García-Pineda, Jesús Campos-García

**Affiliations:** ^1^Laboratorio de Biotecnología Microbiana, Instituto de Investigaciones Químico Biológicas, Universidad Michoacana de San Nicolás de Hidalgo, Morelia, Mexico; ^2^Laboratorio de Interacción Suelo, Planta, Microorganismo, Instituto de Investigaciones Químico Biológicas, Universidad Michoacana de San Nicolás de Hidalgo, Morelia, Mexico; ^3^Laboratorio de Control Traduccional, Instituto de Investigaciones Químico Biológicas, Universidad Michoacana de San Nicolás de Hidalgo, Morelia, Mexico; ^4^Laboratorio de Bioquímica y Biología Molecular, Instituto de Investigaciones Químico Biológicas, Universidad Michoacana de San Nicolás de Hidalgo, Morelia, Mexico

**Keywords:** acyl-CoA synthetase, acyclic terpenes, fatty acids, host colonization, lipopolysaccharides, polyhydroxyalkanoates, biofilm

## Abstract

The pathogenic bacterium *Pseudomonas aeruginosa* possesses high metabolic versatility, with its effectiveness to cause infections likely due to its well-regulated genetic content. *P. aeruginosa* PAO1 has at least six *fadD* paralogous genes, which have been implicated in fatty acid (FA) degradation and pathogenicity. In this study, we used mutagenesis and a functional approach in *P. aeruginosa* PAO1 to determine the roles of the *fadD4* gene in acyclic terpene (AT) and FA assimilation and on pathogenicity. The results indicate that *fadD4* encodes a terpenoyl-CoA synthetase utilized for AT and FA assimilation. Additionally, mutations in *fadD* paralogs led to the modification of the quorum-sensing *las*/*rhl* systems, as well as the content of virulence factors pyocyanin, biofilm, rhamnolipids, lipopolysaccharides (LPS), and polyhydroxyalkanoates. In a *Caenorhabditis elegans in vivo* pathogenicity model, culture supernatants from the 24-h-grown *fadD4* single mutant increased lethality compared to the PAO1 wild-type (WT) strain; however, the double mutants *fadD1/fadD2*, *fadD1/fadD4*, and *fadD2/fadD4* and single mutant *fadD2* increased worm survival. A correlation analysis indicated an interaction between worm death by the PAO1 strain, the *fadD4* mutation, and the virulence factor LPS. Fatty acid methyl ester (FAME) analysis of LPS revealed that a proportion of the LPS and FA on lipid A were modified by the *fadD4* mutation, suggesting that FadD4 is also involved in the synthesis/degradation and modification of the lipid A component of LPS. LPS isolated from the *fadD4* mutant and double mutants *fadD1/fadD4* and *fadD2/fadD4* showed a differential behavior to induce an increase in body temperature in rats injected with LPS compared to the WT strain or from the *fadD1* and *fadD2* mutants. In agreement, LPS isolated from the *fadD4* mutant and double mutants *fadD1/fadD2* and *fadD2/fadD4* increased the induction of IL-8 in rat sera, but IL1-β cytokine levels decreased in the double mutants *fadD1/fadD2* and *fadD1/fadD4*. The results indicate that the *fadD* genes are implicated in the degree of pathogenicity of *P. aeruginosa* PAO1 induced by LPS-lipid A, suggesting that FadD4 contributes to the removal of acyl-linked FA from LPS, rendering modification in its immunogenic response associated to Toll-like receptor TLR4. The genetic redundancy of *fadD* is important for bacterial adaptability and pathogenicity over the host.

## Introduction

*Pseudomonas aeruginosa* is considered pathogenic to humans, insects, nematodes, and animals and possesses high metabolic versatility among bacteria capable of growing on a wide variety of organic compounds (Valentini et al., 2018; Moser et al., 2021). The effectiveness of this species in causing infection and its metabolic versatility may be attributed to its well-regulated genetic content and the accumulation of novel orthologous and paralogous genes in its genome, resulting in genetic redundancy (Stover et al., 2000; Toll-Riera et al., 2016; Panayidou et al., 2020). *P. aeruginosa* is an opportunistic human pathogen capable of causing a wide array of life-threatening acute and chronic infections, particularly in patients with compromised immune defenses. It is the main cause of morbidity and mortality in patients with cystic fibrosis and one of the leading nosocomial pathogens affecting hospitalized patients (Moradali et al., 2017; Rossi et al., 2021). *P. aeruginosa* employs several mechanisms to overcome host defenses, including the production of a wide range of virulence factors that allow its colonization and adaptation in the host, which are finely regulated and dependent on cell-to-cell communication (Lee and Zhang, 2015; Behzadi et al., 2021a; Moser et al., 2021).

The FadD proteins (fatty acid coenzyme A synthetases) involved in fatty acid degradation (FAD) are extensively distributed across all kingdoms of life. Recently, multiple homologs have been identified also as virulence factors in several bacteria such as *Mycobacterium tuberculosis*, *Salmonella enterica* serovar Typhimurium, and *Haemophilus parasuis*; however, its genetic redundancy is uncertain in terms of bacterial pathogenicity (Kang et al., 2010; Feng et al., 2017). In *P. aeruginosa* PAO1, two acyl-CoA synthetases (FadD1 and FadD2) are involved in FA activation, contributing to differential FA degradation and bacterial virulence (Kang et al., 2010). FadD1 has a preference for linear long-chain FA (C_6_–C_14_) and FadD2 for short- and medium-chain FA (C_4_–C_10_). Additionally, other FadD homologs have been identified in *P. aeruginosa*, rendering up to six *fadD* genes (*fadD1–D6*) related to FA degradation (Zarzycki-Siek et al., 2013). The *fadD1* and *fadD2* genes have been linked to virulence through the degradation of phosphatidylcholine (the main component of the lung surfactant), but without causing modifications in the production of virulence factors such as proteases, lipases, and phospholipases (Zarzycki-Siek et al., 2013).

*Pseudomonas aeruginosa fadD1* and *fadD2* are the main genes involved in FA degradation, and *fadD4* (ORF PA1617) is related to acyclic terpene (AT) utilization (Zarzycki-Siek et al., 2013). The ability of *P. aeruginosa* to assimilate acyclic terpenes and leucine/isovalerate as sole carbon and energy sources is well known (Diaz-Perez et al., 2004; Aguilar et al., 2006; Forster-Fromme et al., 2006; Chavez-Aviles et al., 2009; Campos-García, 2010). The acyclic terpene catabolic pathway is considered to consist of four steps: (i) upper oxidation activation pathway, (ii) central AT pathway, (iii) β-oxidation coupling, and (iv) convergence with the leucine/isovalerate pathway (Campos-García, 2010). The genes involved in the AT and leucine/isovalerate pathways are present in the *atuRABCDEFGH* and *liuRABCDE* clusters, respectively; their encoded enzymes have been characterized at the biochemical level, and for a majority of genes, their function in the catabolic pathway is demonstrated (Campos-García, 2010; Díaz-Pérez et al., 2015; Diaz-Perez et al., 2018). The upper pathway of AT degradation involves the oxidation of terpenes such as citronellol, geraniol, or nerol to citronellic and geranic acids (Campos-García and Soberón-Chávez, 2000; Forster-Fromme et al., 2006; Campos-García, 2010). The citronellic or geranic acids produced are then activated by a putative acyl-CoA synthetase, producing citronellyl-CoA or geranyl-CoA, respectively; the corresponding gene remains unknown. The citronellyl-CoA and *trans*-geranyl-CoA metabolites produced in the preceding step are oxidized or isomerized to *cis*-geranyl-CoA, the starting metabolite of the central AT pathway. Therefore, it is suggested that terpenoyl-CoA synthesis is carried out by ATP-dependent acyl-CoA synthetase (Campos-García, 2010). The *atuH* gene has been suggested to encode the acyl-CoA synthase involved in terpenoyl activation (Forster-Fromme et al., 2006); however, this has not been completely clarified.

Fatty acids are important metabolic intermediates and phospholipid components, which are essential for membrane formation. In addition to the energy-expensive FA biosynthesis, most pathogens use and incorporate extracellular FA into their membrane phospholipids and lipopolysaccharides (LPS) (Feng et al., 2017). FadD paralog proteins (FadD1 to FadD6) have been described to be involved in the degree of pathogenesis and virulence mechanism of *P. aeruginosa* PAO1 in a lung mouse infection model, evaluating the modification in the production of virulence factors (such as protease, hemolysin, lipases, phospholipases, and rhamnolipids) (Kang et al., 2010; Zarzycki-Siek et al., 2013).

Multiple virulence factors have been described as inducers or the innate immune responses mediated by polymorphonuclear and macrophage cells (Moser et al., 2021). In the mammalian innate immune responses constituted by leukocyte type-dependent response *via* Toll-like receptors (TLRs), at least 13 TLRs have been identified in mice and humans (McIsaac et al., 2012). TLRs are activated through binding to damage/danger-associated molecular patterns (DAMPs), microbial/microbe-associated molecular patterns (MAMPs), pathogen-associated molecular patterns (PAMPs), and xenobiotic-associated molecular patterns (XAMPs) (Behzadi et al., 2021b).

Toll-like receptors play essential roles in host defense against Gram-negative bacteria (McIsaac et al., 2012; Moser et al., 2021). Thus, TLR1–6 receptors have been associated with the mechanism of recognition of PAMPs such as LPS, flagellin, lipoproteins, pilus, peptidoglycans, etc. in *P. aeruginosa* (Behzadi et al., 2021b; Moser et al., 2021). For example, the TLR2 contributes in the induction of excessive inflammation produced in response to ligands such as lipoproteins, components of the extracellular capsule, and secreted toxins, while that TLR5 response is associated to flagellin. The pathogenesis of *P. aeruginosa* is associated to TLR4 recognizing LPS by binding to the lipid A component on the outer membrane of bacteria (McIsaac et al., 2012; Behzadi et al., 2021b). LPS, as an integral component of the *P. aeruginosa* cell envelope, is important for bacterium–host interactions and has been shown to be a major virulence factor (Lam et al., 2011). In *P. aeruginosa*, the cell surface carbohydrate polymer known as B-band O-antigen, which is a component of LPS, is a major virulence factor, and a wide variety of serotypes exist that differ in the structure of the O-antigen (King et al., 2008; Lam et al., 2011; Lo Sciuto et al., 2019). Genetic determinants have been well characterized, and their involvement in bacterial pathogenicity has been studied; serotype O12 is associated with virulence, multidrug resistance, cystic fibrosis patients, and burn wounds (Pier, 2007; Moradali et al., 2017; Lo Sciuto et al., 2019). Over 50 genes in the *P. aeruginosa* PAO1 genome database are associated with LPS biosynthesis; these clusters are mainly related to antigen O and the core of polysaccharides, but genetic determinants associated with lipid A are poorly described^1^. Some studies describe in cystic fibrosis patients that *P. aeruginosa* produces several different varieties of lipid A on LPS, contributing to the degree of virulence, which is associated to hexa-acylated lipid A, the more potent agonist to TLR4 receptor (McIsaac et al., 2012).

Therefore, the role of FadD proteins in the pathogenicity mechanisms of *P. aeruginosa* and its relation with virulence factor production are of great interest. In this study, a gene mutagenesis and functional approach in *P. aeruginosa* PAO1 were used to determine the roles of *fadD* genes in acyclic terpene and fatty acid catabolism and their contribution to bacterial pathogenicity related with the LPS virulence factor and the immunological response induced.

## Materials and Methods

### Bacterial Strains, Plasmids, and Culture Conditions

Bacterial strains and plasmids used in this work are shown in Table 1. The strains were grown at 30°C in Luria broth (LB) or M9 minimal medium (Sambrook, 2001). For rhamnolipid (RHL) and polyhydroxyalkanoate (PHA) production, *P. aeruginosa* strains were cultured in phosphate-limited protease peptone glucose ammonium salt (PPGAS) medium using glucose as the main carbon source. Solid media were prepared by adding 1.5% agar. Antibiotic concentrations used for *P. aeruginosa* strains were as follows: streptomycin (Sm) 200 μg/ml, gentamicin (Gm) 80 μg/ml, and tetracycline (Tc) 60 μg/ml; for *Escherichia coli* strains, ampicillin (Ap) 100 μg/ml, Gm 20 μg/ml, and Tc 15 μg/ml. Strains were tested for their ability to grow on M9 agar plates supplemented with citronellol, citronellic acid, isovaleric acid (Merck), palm fatty acids, or phosphatidylcholine as sole carbon and energy source after an incubation of 48 h at 30°C.

**TABLE 1:** Strains and plasmids used in this work.

Strains or plasmids	Relevant characteristics	Source
**Strains**		
*P. aeruginosa* PAO1	Spontaneous streptomycin-resistant mutant derived from PAO1 strain.	Wong and Mekalanos, 2000
*fadD1Tn*	PAO1::IS*lacZ*/hah, Tc^R^ (ID 1212), transposon insertion in ORF PA3299, Tc^R^.	Jacobs et al., 2003
*fadD2Tn*	PAO1::IS*lacZ*/hah, Tc^R^ (ID 16715), transposon insertion in ORF PA3300, Tc^R^.	Jacobs et al., 2003
*fadD4Tn*	PAO1::IS*lacZ*/hah, Tc^R^ (ID 21221), transposon insertion in ORF PA1617, Tc^R^.	Jacobs et al., 2003
*fadD2*::*Gm*	PAO1SM mutant obtained by recombination using the ORF PA3300::*Gm*^R^, Sm^R^, Gm^R^.	This work
*fadD4*::*Gm*	PAO1SM mutant obtained by recombination using the ORF PA1617::*Gm*^R^, Sm^R^, Gm^R^.	This work
*PA1618*::*Gm*	PAO1SM mutant obtained by recombination using the ORF PA1618::*Gm*^R^, Sm^R^, Gm^R^.	This work
*fadD1Tn/fadD4*::*Gm*	Double mutant strain derived from PAO*fadD1*^–^, mutated in *fadD4*::*Gm*^R^ gene, Tc^R^ Gm^R^.	This work
*fadD2Tn/fadD4*::*Gm*	Double mutant strain derived from PAO*fadD2*^–^, mutated in *fadD4*::*Gm*^R^ gene, Tc^R^ Gm^R^.	This work
*fadD1Tn/fadD2*::*Gm*	Double mutant strain derived from PAO*fadD1*^–^, mutated in *fadD2*::*Gm*^R^ gene, Tc^R^ Gm^R^.	This work
**Plasmids**		
pGEM-T	Plasmid used for PCR fragments cloning; Ap^R^.	Invitrogen
pG*fadD4*	pGEM-T easy with 1,668-bp DNA fragment containing PA1617 ORF from PAO1, adding *BamH*I and *Hind*III sites in the 5′ and 3′ ends of gene.	This work
pCDFDuet-1	Vector designed for the co-expression of two target genes. MCS, T7 promoter, lac operator, His-tag, and ribosome binding site.	Novagen
pCD*fadD4*	DNA fragment *BamH*I and *Hind*III from pG*fadD4*, containing PA1617 ORF from PAO1.	This work
pG*fadD4::Gm*^R^	pG*fadD4* with gentamycin resistance cassette (900 bp) ligated into *Xho*I site of the PA1617 ORF.	This work
pG*fadD2*	pGEM-T easy with 2,480-bp DNA fragment containing PA3300 ORF from PAO1, adding *BamH*I and *Hind*III sites in the 5’ and 3’ ends of gene.	This work
pG*fadD2::Gm*^R^	pG*fadD2* with gentamycin resistance cassette (900 bp) ligated into *Pst*I site of the *fadD2* gene from PAO1.	This work
pKOK4	pBR325 derived mob + plasmid; Tc^R^, Ap^R^, Cm^R^.	Kokotek and Lotz, 1991
pRK2013	Conjugation helper plasmid, Tra, ColE1 replicon; Km^R^	Figurski and Helinski, 1979
pK*fadD4::Gm*^R^	pKOK4 with *EcoR*I fragment containing *fadD4::Gm*^R^ from pG*fadD4::Gm*^R^.	This work
pK*fadD2::Gm*^R^	pKOK4 with *Hind*III-*BamH*I fragment containing *fadD2::Gm*^R^ from pG*fadD2::Gm*^R^.	This work

### Cloning, Expression, and Purification of Recombinant Proteins

The nucleotide and amino acid sequences analyzed are denoted in the Pseudomonas Genome Project (see Text Footnote 1) as ORFs PA3299, PA3300, and PA1617 (Stover et al., 2000), which were named as *fadD1*, *fadD2*, and *fadD4* genes, respectively (Zarzycki-Siek et al., 2013). The *fadD4* gene was amplified by PCR using PAO1 genomic DNA as template with Platinum Pfx DNA Polymerase (Invitrogen) according to the manufacturer’s instructions. Primers were designed from the PAO1 genome sequence, *fadD4*-forward 5′ GGATCCCATGGTCACTGCAAATCGTCTGCCG 3′ and *fadD4-*reverse 5′AAGCTTTTCGTG CCAGAGCACGGCCTC3′ with the insertion of the restriction sites *Bam*HI and *Hin*dIII. The PCR fragment obtained (∼1.7 kbp) was cloned into the pGEM-T Easy vector to give the plasmid pG*fadD4*. Next, the *fadD4* gene was removed using *Bam*HI and *Hind*III endonucleases (Invitrogen), purified, and ligated into the pCDFDuet-1 vector to obtain the pCD*fadD4* plasmid (Table 1). This plasmid was designed to merge a 6×-histidine tag into the N-terminal end of the FadD4 protein. The plasmids were introduced by electroporation to *E. coli* JM101 cells and subsequently analyzed by digestion with restriction endonucleases and confirmed by DNA sequencing.

Plasmid-transformed *E. coli* JM101 strain was cultured in 100 ml of 2XYT medium for 4 h at 30°C with shaking. Protein expression was induced by adding 0.1 mM isopropyl β-D-1-thiogalactopyranoside (IPTG), and the cells were further incubated for 12 h. Cells were harvested by centrifugation, and bacterial pellets were suspended in 10 ml of buffer A (50 mM Tris-HCl, 500 mM NaCl, pH 7.8) and disrupted by sonication at 4°C. Bacterial lysates were centrifuged for 15 min at 15,000 × *g* at 4°C to obtain cell-free protein extracts. Recombinant proteins were purified from cell-free protein extracts to homogeneity by affinity chromatography according to the TALON^TM^ purification kit protocol (BD Biosciences) with slight modifications (Chavez-Aviles et al., 2009). Protein concentration was quantified using Bradford reagent (Bio-Rad), and proteins were separated and visualized by 10–12% sodium dodecyl sulfate–polyacrylamide gel electrophoresis (SDS-PAGE) (Sambrook, 2001).

### Mutagenesis Procedure

Construction of mutants (Table 1) was done using triparental conjugation as described (Diaz-Perez et al., 2004), among the *P. aeruginosa* PAO1 wild-type strain and the single mutants *fadD1Tn* and *fadD2Tn* using the pRK2013 helper plasmid (Figurski and Helinski, 1979) and the *E. coli* S17.1 strain containing the respective plasmids pKG*fadD2::Gm*^R^ or pKG*fadD4::Gm*^R^ that contain the *fadD2* and *fadD4* genes disrupted as shown (Table 1). Transconjugants were selected on plates with the appropriate antibiotics and submitted to PCR characterization.

### Determination of Acyl-CoA Synthetase Activity

Acyl-CoA synthetase activity was assayed as described by Hume et al. (2009) with slight modifications. The reaction mixture contained Tris buffer (50 mM) pH 8, 12.5 μl MgCl_2_ (100 mM), 50 μl ATP (100 mM), 30 μl substrate (citronellic, geranic, isovaleric, or fatty acids; 10 mM), and 100 μl of free-cell extract (70 μg protein). The mixture was incubated for 5 min at 30°C in a water bath to equilibration temperature. After, 30 μl of coenzyme A (CoA) (20 mM) previously dissolved in Tris buffer (50 mM) pH 8 was added and incubated for 15 min at 30°C in a water bath. Reactions were stopped by adding 450 μl of the ferric chloride reagent, and reaction mixtures were kept on ice for 30 min. The red-purple color absorbance was measured at 540 nm in a UV-Vis spectrophotometer. Neutral hydroxylamine solution, pH 8.0, was prepared by mixing 1 ml hydroxylamine hydrochloride (5 M), 250 μl MilliQ water, and 1.25 ml of KOH (4 M). Ferric chloride reagent was prepared by mixing a 1:1:1 ratio of ferric chloride (0.37 M), trichloroacetic acid (0.02 M), and hydrochloride (0.66 M) (Hume et al., 2009). The molar extinction coefficients of the corresponding fatty acyl hydroxamates were determined experimentally as described (Hume et al., 2009). One unit of enzyme activity is defined as the catalytic activity leading to the formation of 1 nmol of fatty acyl hydroxamate by 1 min. A specific activity is given as unit per milligram of protein.

### Extraction and Analysis of Polyhydroxyalkanoates, Rhamnolipid, and Lipopolysaccharides

For PHA and RHL extraction, *P. aeruginosa* strains were cultured in phosphate-limited PPGAS medium by 48 h of incubation at 37°C. Cells were harvested by centrifugation and washed with 100 mM Tris–100 mM NaCl buffer (pH 7). For the PHA extraction, bacterial pellets were disrupted by sonication and digested with 1.8% sodium hypochlorite for 1 h. After centrifugation, the pellet was washed twice with ethanol and once with acetone (Zhang and Miller, 1992). On the other hand, cell-free supernatants of cultures were used to determine RHL by measuring the rhamnose concentration after acid hydrolysis by the orcinol method (Campos-García et al., 1998).

The lipopolysaccharide fraction on bacterial pellets obtained by centrifugation of cultures was extracted by using two methods, one as described (Latino et al., 2017) and another by the methanol–chloroform method (Mirzaei et al., 2011). LPS was extracted by chloroform–methanol method; briefly, bacterial pellets were recovered from 15 ml of culture in LB medium cultured for 48 h at 37°C at 150 rpm, washed four times with PBS pH 7.4, resuspended in 2 ml of 95% ethanol, vortexed for 1 min, and centrifuged at 2,000 rpm for 10 min; the supernatant was discarded and the pellet was washed with 95% ethanol three times. The pellet was dried and resuspended in 1 ml of 10% EDTA by sonication. Then, 1 ml of methanol–chloroform (1:2) was added, stirred for 2 h, and centrifuged at 2,000 rpm for 10 min; the upper phase (methanol) was discarded and the lower phase (chloroform) is taken and evaporated for the LPS quantification. LPS extraction samples were run on SDS-PAGE resolving gels (12%), and gels were stained by silver staining based on standard protocol (Sambrook, 2001; King et al., 2008). Surface polysaccharides of the LPS isolated were measured by the sulfuric acid-phenol colorimetric method using sucrose and fructose as sugar standards (Brimacombe et al., 2013).

### Determination of Fatty Acid Content From Polyhydroxyalkanoates and Lipopolysaccharides

Fatty acid content on PHA and LPS was determined by quantitation of fatty acid methyl esters (FAME) by gas chromatography–mass spectrometry (GC-MS), submitting the samples to methyl esterification as follows. Briefly, total PHA or LPS was dissolved with chloroform–methanol–water and carried out to methyl esterification for 1 h at 80°C in methanol and H_2_SO_4_ (Campos-García et al., 2018). The FAME were extracted with hexane, samples were suspended in 500 μl of chloroform, and 1 μl was analyzed using GC-MS (GC; Agilent 6850 Series II equipped with MS-5973 and FID detectors), using a Zebron ZB-FFAP column, 30-m length, I.D. 0.25 mm, and film 0.25 mm (Phenomenex) (Campos-García et al., 2018). Sample injection was performed at a splitless mode at a temperature of 280°C and an FID detector temperature of 300°C. The oven temperature was programmed to start at 50°C, maintained in isothermal for 5 min, then increased to 280°C at a rate of 10°C/min, and then isothermal 280°C for 5 min. Compounds were identified by comparison with the mass spectral library (NIST/EPA/NIH, ChemStation, Agilent Technologies Rev. D.04.00 2014). FAME mass profiles were confirmed by comparing with FAME Mix C_4__–_C_24_ standard (Supelco). Quantitation was performed using the relative values of the peak areas in chromatograms obtained by using an FID detector. Results correspond to means of triplicate assays of PHA and LPS samples.

### Determination of Other Virulence Factors and Survival of *Caenorhabditis elegans*

Pyocyanin and quorum sensing (QS) AHL-dependent assays were determined in the cell-free supernatant fraction of *P. aeruginosa* cultures grown in LB medium at 37°C for 24 and 48 h. Pyocyanin was extracted from 1 ml of the cell-free supernatant mixed with 1 ml of chloroform, and the organic fraction was collected and mixed with 1 ml 0.2 N HCl; subsequently, the organic fraction was separated, and the absorbance was measured at 520 nm in a spectrophotometer (Gonzalez et al., 2017). The QS AHL-dependent assay was performed using the *E. coli* JM109 reporter strain transformed in the pSB1075 plasmid, which contains the transcriptional fusion *plasRlasIRhlI:luxCDABE* and produces luminescence in response to 3-oxo-C_12_-HSL (Winson et al., 1998). Briefly, the *E. coli* JM109 (pSB1075) strain was grown overnight in LB liquid medium at 37°C with shaking. Then, the culture was diluted with fresh LB medium to obtain an optical density (OD) of 0.1 at 600 nm (OD_600_), and 200 μl was distributed in each well of a 96-well culture plate and incubated for 2 h at 37°C. Subsequently, 50 μl of cell-free *P. aeruginosa* culture supernatants were added, followed by incubation at 37°C with slight shaking. Both the OD_600_ and luminescence were determined at 4 h using a ChemiDoc MP imaging system (Bio-Rad) (Gonzalez et al., 2017).

For quantification of biofilm, cultures of *P. aeruginosa* strains were adjusted to OD_600_ = 0.1 optical density with LB medium, and 100-μl aliquots of diluted cultures were collocated into 96-well microtiter plates and incubated at 37°C for 48 h. Culture media were eliminated, and biofilm quantification was done by dyeing the 96-well plate with crystal violet and measuring the absorbance of samples in the plate at 550 nm (Coffey and Anderson, 2014).

Survival assays using *C. elegans* Bristol N2 and CL2166 dvIs19 [pAF15 (Pgst-4::GFP::NLS)] worms (provided by the Caenorhabditis Genetics Center, University of Minnesota) were synchronized by hypochlorite isolation of eggs from gravid adults (Stiernagle, 2006). Briefly, L1 larvae were transferred onto nematode growth medium plates seeded with the *E. coli* OP50 strain previously grown on the plates as a food source and were incubated at 18°C for 4–5 days until they reached the young adult phase. Worms were incubated either with cell-free medium collected from bacterial cultures grown for 24 and 48 h in LB medium (slow-killing assay) or bacterial strains (fast-killing assay). For each experiment, 20–30 worms were placed, and plates were incubated at 25°C and scored for life at 6, 12, 24, and 48 h. For statistical purposes, at least three replicates per trial were carried out. *E. coli* OP50 culture supernatant was used as a negative control. *C. elegans* worms were considered dead when they stopped moving and did not respond to a nudge with a platinum wire. The fluorescence microscopy images obtained were taken using a fluorescent and phase contrast inverted microscope (Nikon Eclipse TE300) with Plan Fluor 4× and 10× dry lenses, and an AmScope ML300 3.1-MP digital color camera.

### Western Blot Analysis

Cell-free protein extracts were used for immunoblot assays. Thirty micrograms of total protein previously quantified was mixed with 10 μl of denaturing buffer (Tris-HCl 0.06 M, pH 6.8, 5% glycerol, 4% SDS, 4% β-mercaptoethanol, and 0.0025% bromophenol blue) for 5 min at 95°C in a boiling water bath. Samples were run in SDS-PAGE at 10%. The gels in one side were Coomassie blue stained and the other gels were transferred to polyvinylidene difluoride (PVDF, Millipore) membrane. For immunodetection, membranes were blocked using dry milk in TBS-T (Tris-HCL 10 mM; NaCl 0.9%; and 0.1% Tween-20, pH 7.8) and blotted with the respective antibody. The FadD4 protein was detected using anti-His-tag antibody (Invitrogen) and the anti-mouse–HRP conjugate as the secondary antibody (Santa Cruz Biotechnology) as indicated by the provider. His-tag SDS-PAGE protein standard (Invitrogen) was used as molecular markers. On the other hand, the antibodies IL-8 sc-376750, IL-1β sc-32294, and IL-1β sc-52012 (Santa Cruz Biotechnology) were blotted in a blocking medium at 1:5,000 dilution overnight at 4°C with light shaking. After washing, membrane was incubated with the secondary antibody, Goat anti-Mouse IgG HRP conjugate (Bio-Rad), in a blocking medium at 1:10,000 dilution for 2 h at 4°C. Membranes were washed twice with TBS-T and developed using hydrogen peroxide and SuperSignal West Pico Luminol, exposing in ChemiDoc^TM^ MP System (Bio-Rad). At least three independent assays were conducted, and representative images are shown. Band intensities in images were quantified using the ImageJ1 software (NIH Image).

### Rat Exposition to Lipopolysaccharides

Twenty four healthy Wistar rats (≥250 g body weight) were housed in rodent plastic boxes and placed in a temperature/humidity/light-controlled chamber set at 22 ± 1°C, 12:12-h light/dark cycle, and unlimited access to food and water before treatment. Animal handling, feeding, and care were done by trained personnel following the National Institutes of Health Guide for the care and use of laboratory animals. This research was also approved by the Institutional Committee for Use of Animals of the I.I.Q.B/Universidad Michoacana de San Nicolás de Hidalgo in agreement with NOM-062-ZOO-1999, Ministry of Agriculture, Mexico. Lipopolysaccharides extracted from *P. aeruginosa* PAO1 strains were dissolved in sterile 0.9% sodium chloride and injected intraperitoneally at a dose of 50 μg/kg (Kozak et al., 2006). Body temperature was monitored every 15 min for 4 h, and finally, the animals were euthanized by receiving an overdose of sodium pentobarbital. Blood was collected, white blood cell count was examined by microscopic observation, and serum was separated for interleukin determination by western blot.

### Statistical Analysis

For correlation data analysis, data obtained in all assays were analyzed by utilizing response variables (strains) vs. numerical data for each assay (cases) using the STATISTICA software (Data Analysis Software System 8.0; Stat Soft Inc.). Other data were statistically analyzed using GraphPad Prism 6.0 software (GraphPad Software Inc.).

## Results and Discussion

### Genomic Context of the *fadD4* Gene in *P. aeruginosa* PAO1

**FIGURE 1:**
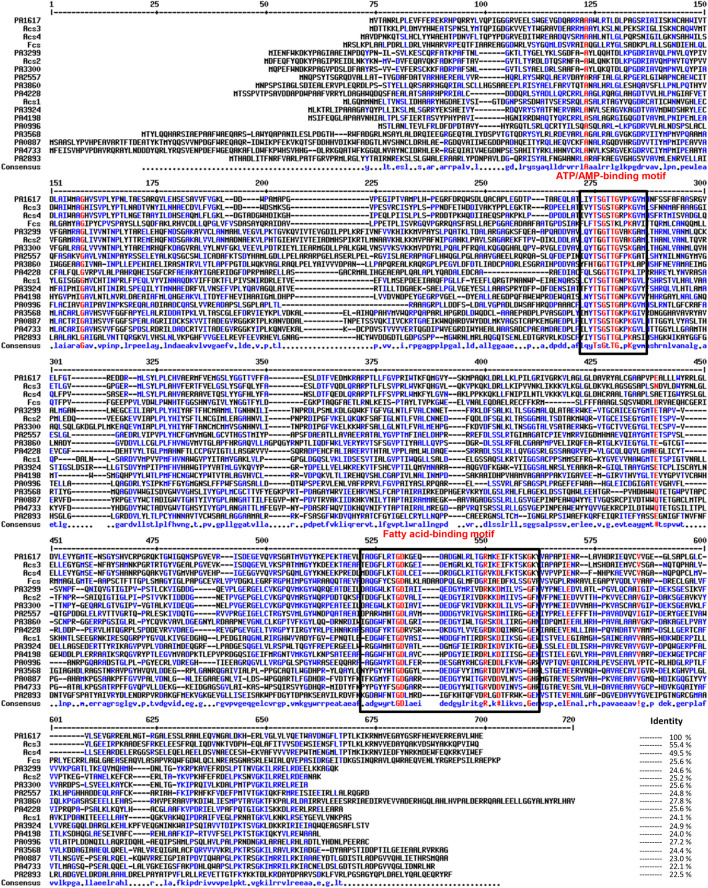
Multiple sequence alignment of acyl-CoA synthetases from *Pseudomonas aeruginosa* PAO1. The ATP/AMP and fatty acid binding motifs are indicated by boxes. The accession numbers correspond to the following proteins used in the analysis: Acs1 (EF219372), Acs2 (EF219373), Acs3 (EF219374), and Acs4 (EF219375) from *Marinobacter hydrocarbonoclasticus* DSM8798 and feruloyl-CoA synthetase (Fcs; CAD60268) from *Pseudomonas fluorescens* BF13; from *P. aeruginosa* PAO1 genome, FadD1 (PA3299), FadD2 (PA3300), FadD3 (PA3860), FadD4 (PA1617), FadD5 (PA2893), FadD6 (PA3924), PA0887, PA0996, PA2557, PA3568, PA4198, PA4228, and PA4733. Multiple sequence alignments with hierarchical clustering were made using MultAlin software (Corpet, 1988).

Multiple homologs of *fadD* genes have been identified in *P. aeruginosa* PAO1, which are involved in FA catabolism and are also related to virulence; however, its genetic redundancy is uncertain in terms of bacterial pathogenicity (Kang et al., 2010; Zarzycki-Siek et al., 2013). In this study, an exhaustive bioinformatics analysis indicated that the PAO1 genome contains approximately 30 proteins paralogous to FadD1/FadD2 (proteins with 500–700 amino acids showing at least the AMP-binding protein motif) (Supplementary Table 1). The ORF PA1617 in the *P. aeruginosa* PAO1 genome, which was designated as *fadD4* gene in an earlier report (Zarzycki-Siek et al., 2013), encodes a protein of 555 residues with a predicted molecular weight of 61.4 kDa (Pseudomonas Genome Database, PGD). The PA1617-encoded protein contains the characteristic domains for ATP/AMP binding found in the fatty acyl-AMP ligase/fatty acyl-CoA synthetases (YTSGTTGVPKGA-N117-EVYGMTE) and the fatty acid-binding domain (DGFLRTGDKGEQDADGNLRLTGRMR), which indicates that it belongs to the family of fatty acyl-CoA synthetases (FadDs) involved in fatty acid degradation (Figure 1).

**FIGURE 2:**
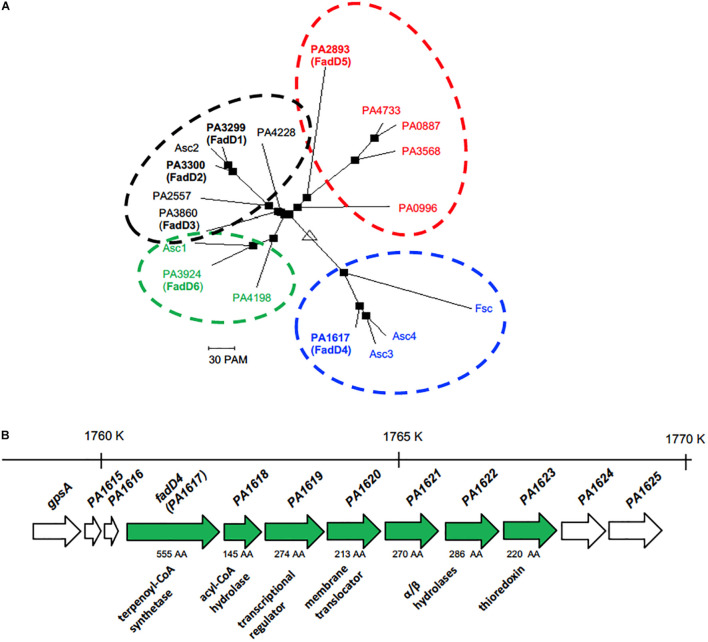
Phylogenetic and genetic contexts of the PA1617 ORF in *P. aeruginosa* PAO1 genome. **(A)** Phylogeny tree of the alignment shown in Figure 1. **(B)** Location of the PA1617 (FadD4) gene cluster in the *P. aeruginosa* PAO1 genome. The numbers and orientation of ORFs correspond to their locations in the PAO1 genome (http://www.pseudomonas.com). The genomic region is indicated with numbers above each gene cluster; the length of genes based on the number of amino acids is indicated with a number below the arrows. Solid arrows denote the genes corresponding to the cluster. The gene sizes on ORFs are not on the same scale.

*Pseudomonas aeruginosa* PAO1 contains multiple homologous proteins that show both the characteristic ATP/AMP binding and fatty acyl-CoA domains, suggesting that they could be involved in AMP activation of multiple substrates and participate in multiple metabolic pathways, in addition to FA oxidation (Zarzycki-Siek et al., 2013). In this context, our BLAST protein sequence analysis showed 12 highly conserved paralogous proteins, containing the FadD protein domains. The PA1617 ORF from *P. aeruginosa* shared an amino acid sequence identity of 24.6% with FadD1 (PA3299), 25.2% with FadD2 (PA3300), 27.8% with FadD3 (PA3860), 22.5% with FadD5 (PA2893, also called AtuH), and 24.9% with FadD6 (PA3924). In addition, it shared a similar sequence identity with the remaining paralogs: 23% with PA0887, 27.2% with PA0996, 24.8% with PA2557, 24.4% with PA3568, 24% with PA4198, 25.6% with PA4228, and 22.1% with PA4733 (Figure 1). Interestingly, FadD4 from *P. aeruginosa* showed good sequence identity with homologous proteins from other bacterial species, such as the feruloyl-CoA synthetase (Fcs) from *Pseudomonas fluorescens* BF13 (Calisti et al., 2008) (25.6% identity) and the isoprenoyl-CoA synthetases Acs1 and Acs2 from *Marinobacter hydrocarbonoclasticus* DSM8798 strain (24.1 and 25.2%, respectively) (Holtzapple and Schmidt-Dannert, 2007). However, the highest identities and nearest phylogenetic relationships of the PA1617 ORF were observed with the Acs3 and Acs4 proteins from *M. hydrocarbonoclasticus* DSM8798, showing 55.4 and 49.5% identity, respectively, and with the Fsc protein from *P. fluorescens* BF13 (Figures 1, 2A). It has been reported that *M. hydrocarbonoclasticus* DSM8798 contains several AMP-forming long-chain acyl-CoA synthetases (Acs1–Acs4), which are involved in the CoA activation of fatty acids and isoprenoid fatty acids required for isoprenoid wax ester synthesis when the bacterium is grown on phytol (Holtzapple and Schmidt-Dannert, 2007).

In the resulting phylogenetic tree, four clusters can be clearly observed: FadD1, FadD2, and FadD3 are grouped in the same cluster with the paralogous ORF PA4228 and the isoprenoyl-CoA synthetase (Asc2) from *M. hydrocarbonoclasticus* DSM8798, but FadD5 and FadD6 are located outside the cluster (Figure 2A). FadD5 was grouped with the PA0887, PA0996, PA3568, and PA4733 ORFs, while FadD6 clustered with the PA4198 ORF and the Acs1 protein from *M. hydrocarbonoclasticus* DSM8798. Interestingly, PA1617 (FadD4) was grouped external to others with the Asc3 and Asc4 proteins from *M. hydrocarbonoclasticus* DSM8798 and feruloyl-CoA synthetase (Fcs) from *P. fluorescens* BF13 (Figure 2A). These data allowed us to hypothesize that the ORF PA1617 from *P. aeruginosa* PAO1, named *fadD4* gene, could encode the main functional terpenoyl-CoA synthetase from *P. aeruginosa*. Interestingly, bioinformatics findings indicated a distant phylogenetic relationship of the FadD4 with the FadD1/D2/D3/D5/D6 paralogs, as codon usage comparison between *fadD4* (PA1617) and *fadD1* genes also showed significant differences in bias and frequency of rare used codons (Supplementary Figure 1), suggesting that the *fadD4* gene might have been acquired by horizontal transfer from other bacterial species and, consequently, could exhibit a different biological function.

In addition, our computational analysis suggests that a possible operon may be constituted by the ORFs PA1617 to PA1623 (Figure 2B). The putative function of PA1618 is related to the 4-hydroxybenzoyl-CoA thioesterase enzyme, which could play roles in thioester hydrolysis in fatty acid metabolism. The following PA1619 and PA1620 ORFs share a sequence similarity with transcriptional regulators of the AraC family and transmembrane solute translocators, respectively. PA1621 and PA1622 are homologs of the alpha/beta hydrolase family, such as pimeloyl-ACP methyl ester carboxylesterase, and finally, the PA1623 ORF is homologous to the thioredoxin-like superfamily.

### The *fadD4* Gene Is Implicated in Acyclic Terpene and Fatty Acid Assimilation

The functions of the paralogs *fadD1–D6* in the *P. aeruginosa* PAO1strain have been described to encode enzymes for fatty acyl-CoA activation, recognizing FA substrates of different chain lengths; however, its genetic redundancy, bacterial metabolism, and virulence are not clearly known yet. The *fadD1* and *fadD2* genes in the *P. aeruginosa* PAO1 strain have been described to encode acyl-CoA synthetases that recognize FA substrates of different chain lengths and are also related with phosphatidylcholine assimilation (Zarzycki-Siek et al., 2013). However, the functions of the paralogs FadD3–FadD6 are not clearly known yet. A mutagenesis approach and a functional study let us delve deep in elucidating these implications. Previous mutagenesis studies in our laboratory on the *P. aeruginosa* PAO1 strain showed that the ORF PA1617 mutation impairs the ability to grow on acyclic terpenes such as citronellol (Campos-García, 2010). Therefore, the ability of some of the *fadD* mutants to grow on acyclic terpenes was evaluated.

The *fadD1Tn* and *fadD2Tn* mutants, which were obtained by transposon insertion into the *fadD1* (ORF PA3299) and *fadD2* (ORF PA3300), respectively (Table 1), were capable of growing on solid minimal medium supplemented with (as sole carbon source) *n*-octanol, leucine, or isovaleric acid, similar to the wild-type (WT) strain, but their growth was impaired on terpenes such as citronellol, geraniol, citronellic acid, or geranic acid (Table 2). In agreement, the transposon-obtained mutant *fadD4Tn* (transposon mutant in ORF PA1617) showed a similar ability to grow on terpenes as *fadD1* and *fadD2* mutants (Table 2). Interestingly, a mutant obtained by genetic recombination of a disrupting antibiotic cassette into the ORF PA1617 (*fadD4*::*Gm*) showed a clear impairment of growth on acyclic terpenes (Table 2). This result indicates that both the *fadD1* and *fadD2* genes may be involved in acyclic terpene assimilation, being that both genes are not essential, probably due to genetic redundancy. This observation is in agreement with the ability of FadD homologs to activate acyclic terpenes of a wide range of chain lengths, suggesting that the *P. aeruginosa* FadD1 and FadD2 enzymes could also activate acyclic terpenoic acids (Kang et al., 2010; Zarzycki-Siek et al., 2013).

**TABLE 2:** Growth analysis of *P. aeruginosa* strains in different compounds.

Strain	Compound						
	Octanol	Citronellol	Geraniol	Citronellic	Geranic	Leucine	Isovaleric
PAO1 (WT)	+++	+++	++	+++	+++	+++	+++
*fadD1Tn*	+++	++	+	++	++	+++	+++
*fadD2Tn*	+++	++	+	++	++	+++	+++
*fadD1Tn/fadD2::Gm*	+++	++	++	+++	++	+++	+
*fadD4Tn*	+++	++	++	++	++	+++	+++
*fadD4::Gm*	+++	+	− (+/−)	+	+	+++	++
*PA1618::Gm*	+++	+	− (+/−)	+	+	+++	++
*fadD1Tn/fadD4::Gm*	+++	− (+/−)	− (+/−)	− (+/−)	− (+/−)	+++	+++
*fadD2Tn/fadD4::Gm*	+++	− (+/−)	− (+/−)	− (+/−)	− (+/−)	+++	++

*The strains were inoculated in M9 plates supplemented with the compounds indicated as the only carbon source and incubated for 48 h at 30°C. +++, extensive growth; ++, intermediate growth; +, poor growth; +/−, poor growth after 4 days; −, no growth. Tn, Tn5 mutants (::Tn5ISlacZ/hah, tet^R^) were obtained by Jacobs et al. (2003). Single and double mutants in fadD genes (::Gm^R^) were obtained by gene disruption using the Gm^R^ cassette by homolog recombination.*

To verify this assumption, a double mutant of the *fadD1* and *fadD2* genes was constructed. The transposon-obtained mutant *fadD1Tn* was mutagenized with the *fadD2* gene, utilizing the *fadD2* PCR product obtained by disruption with a cassette of gentamicin resistance (*fadD2::Gm*) (Table 1). Interestingly, the double mutant *fadD1Tn/fadD2::Gm* was capable of growing on acyclic terpenes, similar to their single mutants (Table 2). This result not only confirms that, in the *P. aeruginosa* PAO1 strain, the *fadD1* and *fadD2* genes are not essential for acyclic terpene assimilation but also confirms the existence of homolog genes that encode to a terpenoyl-CoA synthetase.

The participation of *fadD4* in acyclic terpene catabolism and its genetic redundancy were elucidated by the construction of double mutants in combination with *fadD1* and *fadD2* mutants, by utilizing the disrupted PA1617 ORF with a gentamicin resistance cassette (*fadD4::Gm*) (Table 1). The *fadD1Tn/fadD4::Gm* and *fadD2Tn/fadD4::Gm* double mutants were unable to grow on acyclic terpenes (citronellol, geraniol, citronellic acid, and geranic acid); however, these mutants were capable to grow on leucine and isovaleric acid (Table 2). In contrast, as mentioned above, the *fadD1Tn/fadD2::Gm* double mutant grew on the terpenes tested.

**FIGURE 3:**
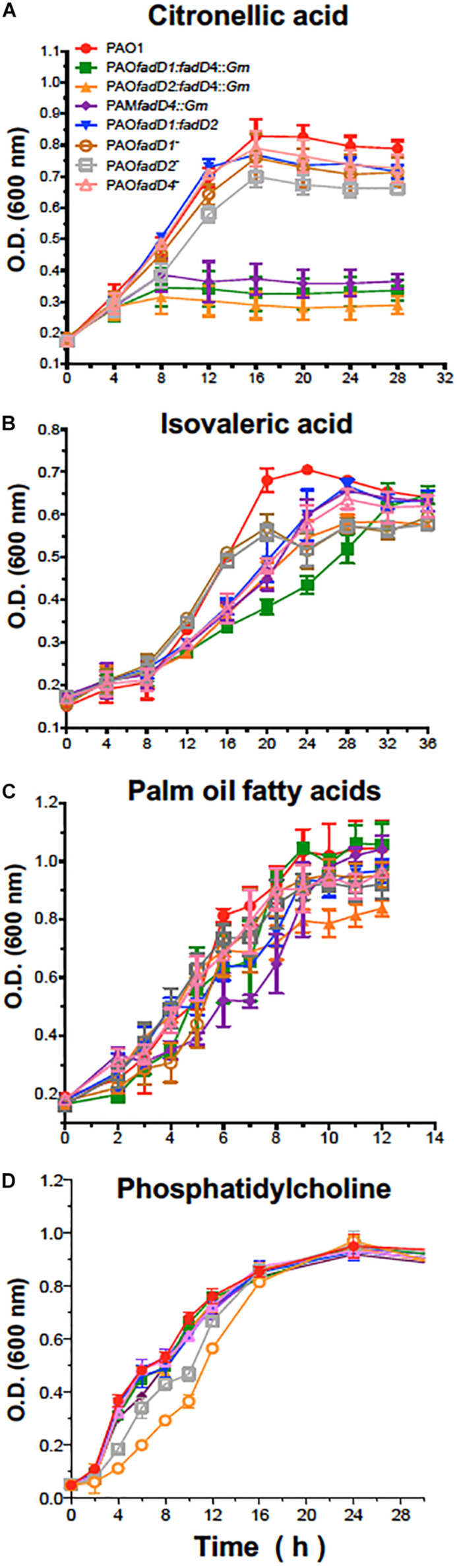
Bacterial growth of *P. aeruginosa* PAO1-derived strains. Cultures were grown on M9 minimal medium with 0.1% citronellic acid **(A)**, 0.1% isovaleric acid **(B)**, 0.1% oil fatty acids **(C)**, or 0.4% phosphatidylcholine **(D)** at 30°C with shaking. Results are shown as means ± SE of triplicate experiments.

Additionally, the disrupted *fadD4::Gm* single mutant and the double mutants *fadD1Tn/fadD4::Gm* and *fadD2Tn/fadD4::Gm* were unable to grow efficiently on liquid M9 medium with citronellic acid as the sole carbon source, whereas the double mutant *fadD1Tn/fadD2::Gm* and the single mutants *fadD1Tn* and *fadD2Tn* did not show any significant difference in growth compared to the WT strain (Figure 3A). Although the growth on isovaleric acid was slightly affected in some mutants, they were capable of growing as well as the WT strain, with palm oil FA and phosphatidylcholine as carbon sources (Figures 3B–D). These results indicate that in *P. aeruginosa* PAO1, the *fadD4* gene is essential for the assimilation of acyclic terpene, but not for leucine/isovalerate, suggesting that *fadD4* encodes for the terpenoyl-CoA synthetase, further confirming the existence of genetic redundancy in the *fadD* genes.

In this study, the mutant *fadD4Tn*, whose transposon is located in the first third of the PA1617 ORF, was deficient but not incapable to grow on acyclic terpenes. In contrast, the growth of *PA1618*::*Gm* mutant obtained by disruption of the PA1618 ORF was severely impaired on terpenes as sole carbon sources (Table 2). These growth phenotypes may be explained by the *fadD4Tn* mutant not causing polar effects over downstream genes contained in the putative operon identified (Figure 2), allowing the genetic expression of the downstream genes; in contrast, the gene disruption in the *fadD4*::*Gm* and *PA1618*::*Gm* mutants prevents the expression of downstream genes due to the presence of a transcription terminator sequence in the resistance cassette. These effects suggest that the downstream genes (ORF PA1618 to PA1623) could be involved in subsequent steps of acyclic terpene and fatty acid degradation; in addition, mutations in the *fadD4* and PA1618 ORFs by gene disruption reveal that the *fadD4* operon plays an essential role in acyclic terpene assimilation, suggesting that other ORFs that constitute the operon could also be involved; however, further investigation is required to elucidate their function.

### Enzymatic Activity of the Recombinant FadD4 Protein

**FIGURE 4:**
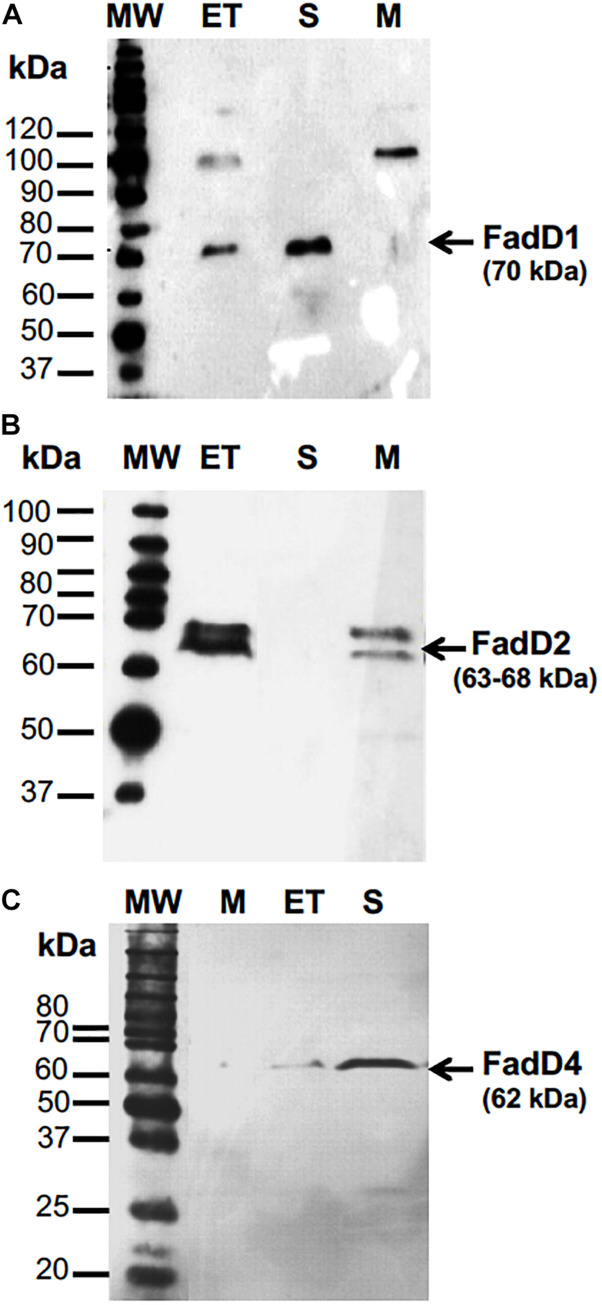
The expression of recombinant FadD1, FAdD2, and FadD4 proteins of *P. aeruginosa* PAO1 in *E. coli*. Purification and western blot analysis of **(A)** FadD1, **(B)** FadD2, and **(C)** FadD4. Western blot detection used anti-His antibody. MW, molecular mass marker; ET, cell-free protein extract; S, soluble fraction; M, membrane fraction. Protein molecular weight markers in kilodaltons are indicated.

To study the function of the FadD4 protein, its expression level and enzymatic activity were determined. The *fadD4* gene (PA1617) was amplified by PCR as a 1,668-bp DNA fragment, and the *fadD1* and *fadD2* genes were also subcloned separately into an expression vector designed to merge a 6-histidine tag into the N-terminal ends of the FadD proteins and introduced by electroporation into *E. coli* JM101, as described in Section “Materials and Methods.” Cell-free protein extracts of cultures induced with IPTG were monitored for recombinant protein expression on SDS-PAGE gels. After purification by affinity chromatography, we observed a ∼70-kDa protein for the *fadD1* gene product, two bands of ∼63–68 kDa for the *fadD2* gene product, and a ∼62-kDa protein band corresponding to the *fadD4* gene product, all of which were confirmed by western blot using anti-His antibody (Figure 4). Additionally, the FadD4 and FadD1 proteins were located in the soluble fraction, while the recombinant FadD2 protein was mainly located in the membrane fraction (Figure 4). These data suggest that both FadD1 and FadD4 could be cytoplasmic or extracellular proteins, while the FadD2 protein could be associated with the membrane.

The recombinant FadD4 protein was purified by affinity chromatography, and the dialyzed fraction was used for enzymatic activity determination. Using this procedure, we were unable to isolate a stable fraction with activity for subsequent enzyme characterization; therefore, cell-free protein extracts of cultures of *E. coli* BL21(DE3) transformed with respective plasmids were used to determine the acyl-CoA synthetase activity following a previously described method (Kang et al., 2010).

Protein extracts of *E. coli* overexpressing the recombinant FadD4 protein showed similar levels of enzymatic activity with different substrates, such as citronellic acid (418 μmol/min/mg), geranic acid (336 μmol/min/mg), and long-chain fatty acids (C_12__–_C_18_ mix; 358 μmol/min/mg), and reduced activity with isovaleric acid (148 μmol/min/mg) (Table 3). These results indicate that the FadD4 protein in *P. aeruginosa* PAO1 encodes an acyl-CoA synthetase capable of using acyclic terpenoic acids, fatty acids, and isovaleric acid as substrates. This finding indicates that, like FadD1 and FadD2, FadD4 is active on a wide range of substrates. However, the growth phenotype indicates that FadD4 corresponds to the terpenoyl-CoA synthetase, which is preferentially required for acyclic terpene assimilation, suggesting that FadD1/D2 versus FadD4 is subject to different genetic regulation processes depending on the substrate and growth conditions.

**TABLE 3:** Acyl-CoA synthetase activity in cell-free extracts of *E. coli* expressing the *fadD4* gene from *P. aeruginosa* PAO1.

Substrate	Acyl-CoA synthetase (μmoles/min × mg protein)	
	*E. coli*	*E. coli* (pCD*fadD4*)
Citronellic acid	30 ± 8	418 ± 132*
Geranic acid	0 ± 4	336 ± 57*
Isovaleric acid	8 ± 4	148 ± 50
Long-chain fatty acids	25 ± 5	358 ± 92*

Recombinant FadD4 enzyme was expressed in Escherichia coli BL21(DE3) strain, and cell-free extracts were used for determination of acyl-CoA synthetase specific activity. The activity was determined using 10 mM of substrates, 10 mM ATP, 10 mM Mg^2+^ in phosphate buffer, pH 7.0, and 30°C by 30-min reaction (Kang et al., 2010). Values of means ± SD were analyzed by multiple t-test-one; significant differences with respect to basal activity are indicated (*), n = 3, p < 0.05.

### The *fadD4* Mutation Modifies the Production of Virulence Factors

FadD paralog proteins (FadD1 to FadD6) have been described to be involved in the degree of pathogenesis and virulence mechanism of *P. aeruginosa* PAO1. Regarding the FadD1 and FadD2 members of the FA regulon, FadD2 mutants exhibited a decreased production of lipase, protease, rhamnolipids, and phospholipase; both mutants showed noticeable deficiencies in assimilating FAs and phosphatidylcholine, which translated to a decreased infection capability in a mouse lung model (Kang et al., 2010). FadD1 plays a dominant role in FA metabolism, whereas FadD2 is activated only when FadD1 is inactivated, suggesting different levels of regulation. High genetic redundancy in *P. aeruginosa* increases the uncertainty about the function of FadD enzymes. However, a link between FA degradation, nutrient metabolism, and the expression of virulence factors is known. FadD1 and FadD2 mutants showed decreased competition levels in lung colonization, with the FadD2 mutant and the double mutant FadD1D2 being more deficient than the FadD1 mutants (Kang et al., 2010); however, the authors described that a significant modification in the production of virulence factors (such as protease, hemolysin, lipases, phospholipases, and rhamnolipids) was not observed, except in the multiple-mutant *fadD1D2D3D4D5D6*, which showed diminished levels (Kang et al., 2010; Zarzycki-Siek et al., 2013). As the multiple mutagenesis procedure may be associated with pleiotropic effects, we considered to re-evaluate the FadDs’ roles in the pathogenesis of *P. aeruginosa*; therefore, a construction of novel mutants in the *fadD1*, *fadD2*, and *fadD4* genes by means of a different methodology was carried out to elucidate whether they are involved.

The *las/rhl* systems of QS have been described to hierarchically control the production of major virulence factors in *P. aeruginosa* (Lee and Zhang, 2015). Thus, we evaluated the ability of the culture supernatants of the *P. aeruginosa* strains (PAO1 WT, *fadD1Tn*, *fadD2Tn*, *fadD4::Gm*, *fadD1Tn/fadD2::Gm*, *fadD1Tn/fadD4::Gm*, and *fadD2Tn/fadD4::Gm*) to induce bioluminescence in the QS reporter strain, using as a negative control the QS system double mutant PAO1-JM2 (*lasI^–^/rhlI^–^*) previously described (Li et al., 2007).

**FIGURE 5:**
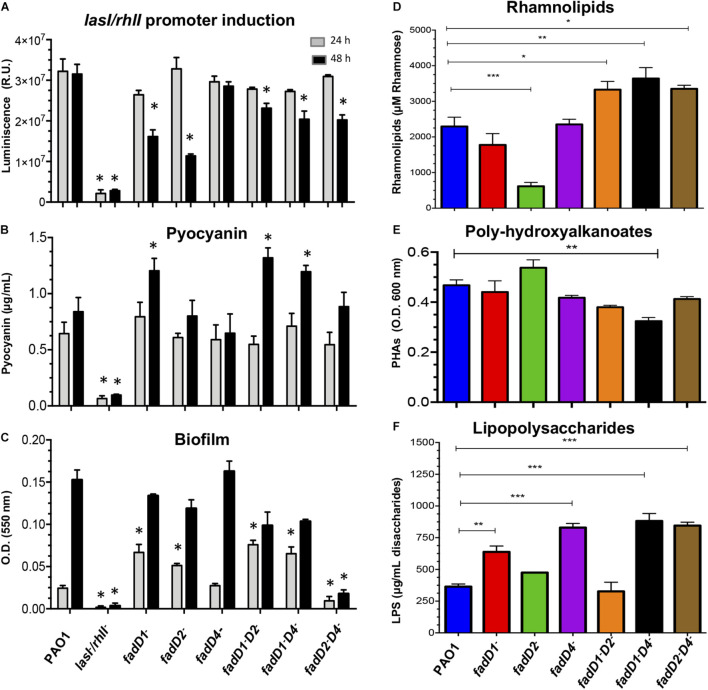
Determination of virulence factors in the cultures of *P. aeruginosa* PAO1 *fadD* mutants. **(A–C)** Determination of virulence factors in the cell-free supernatants of *P. aeruginosa* PAO1 strains as described in Section “Materials and Methods.” **(A)** Induction of the *las/rhl*-dependent biosensor, determined by bioluminescence generation. The assay was performed using the *E. coli* JM109 reporter strain harboring the pSB1075 plasmid, which produces luminescence in response to 3-oxo-C_12_-HSL. **(B)** Pyocyanin, **(C)** biofilm formation, **(D)** rhamnolipids, **(E)** polyhydroxyalkanoates, and **(F)** lipopolysaccharides. **(D–F)** Cell-free supernatants of 48-h grown cultures. Bars represent the mean ± SE of three independent experiments. One-way ANOVA was performed, with Bonferroni *post hoc* test; *n* = 3. Values for SE (*P* < 0.05*, <0.001**, <0.0001***) are shown.

The cell-free supernatants of cultures obtained at 24 h from all the single and double mutants were able to induce a QS-dependent bioluminescence of the reporter strain, with similar intensity as the WT PAO1 strain supernatant (Figure 5A). However, when supernatants of cultures grown for 48 h were tested, the *fadD1Tn* and *fadD2Tn* mutants showed a diminished luminescence intensity compared to the WT PAO1 strain, but the luminescence level induced by supernatants from the *fadD4::Gm* mutant did not change. Instead, the double mutants *fadD1Tn/fadD2::Gm*, *fadD1Tn/fadD4::Gm*, and *fadD2Tn/fadD4::Gm* showed a diminished luminescence intensity compared to the WT strain. These findings indicate that the mutations in *fadD1* and *fadD2*, but not in *fadD4*, affected the AHL-dependent *las/rhl* QS response, suggesting that the consequent modifications in the production of AHL-dependent virulence factors occurred on *fadD1* and *fadD2* mutants but did not occur in the *fadD4::Gm* mutant.

Virulence factors such as pyocyanin (Lau et al., 2004) and biofilm (Vital-Lopez et al., 2015; Thi et al., 2020) are important in bacterial–host colonization, and their production are dependent of QS regulation systems. Biofilm is a life mode that consists of microbes contained in slow-growing microcolonies embedded in a protective biopolymer matrix, which confers highest levels of resistance to antibiotics and the immune system. Biofilm is a common cause of persistent infections, being a key pathogen of chronic infections in the lungs of cystic fibrosis and immunocompromised patients (Moser et al., 2021). In *P. aeruginosa*, a biofilm is a protective structure for bacterial growth, essential for survival in nosocomial environments, and it may also maintain a persistence of the inflammatory response in chronic bacterial infections, favored by the excretion of diverse virulence factors; however, the degree of pathogenicity, expression of virulence factors, or resistance to antibiotic by bacteria cannot always be associated with the ability to form biofilms (Gajdács et al., 2021).

Findings showed that pyocyanin production in mutants was similar to that in the supernatants after 24-h growth; however, in supernatants at 48 h, a significant increment of ∼60% was observed in the *fadD1Tn* single mutant, as well as in the double mutants *fadD1Tn/fadD2::Gm* and *fadD1Tn/fadD4::Gm* (Figure 5B). The amount of biofilm formed was significantly increased in the mutants *fadD1Tn*, *fadD2Tn*, *fadD1Tn/fadD2::Gm*, and *fadD1Tn/fadD4::Gm* with supernatants after 24-h growth. However, it remained similar between the WT and the *fadD4::Gm* mutant, while in the double mutant *fadD2Tn/fadD4::Gm*, the level of biofilm was diminished (Figure 5C). Interestingly, in supernatants from bacterial cultures grown for 48 h, none of the mutants showed significant differences in biofilm production compared to the WT strain, except for the double mutant *fadD2Tn/fadD4::Gm*, in which biofilm production was severely affected (Figure 5C). These results indicate that the *fadD1/2/4*-encoded proteins in *P. aeruginosa* PAO1 play important roles in biofilm production, probably associated with FA catabolism and energy availability.

Other QS-regulated virulence factors that are essential for host colonization of *P. aeruginosa*, which contain lipidic components in their structures, are RHL, PHA, and LPS (Pier, 2007; Reis et al., 2011; García-Reyes et al., 2020). In our study, we found that the RHL content was increased in the cell-free supernatants obtained at 48 h from cultures of the double mutants but was significantly impaired in the *fadD2Tn* mutant (Figure 5D).

In addition, it has been described that compounds that contain lipidic components such as PHA can be redirected from carbon storage to RHL synthesis (Gutiérrez-Gómez et al., 2019) and, in consequence, may increase the virulence and colonization capacity of the bacterium. Our results showed that the PHA content was unmodified in all *P. aeruginosa* strains, except in the double mutant *fadD1Tn/fadD4::Gm*, in which it was decreased by approximately 20% (Figure 5E).

Interestingly, the LPS content was significantly increased in the *fadD1Tn* and *fadD4::Gm* mutants, as in the double mutants *fadD1Tn/fadD4::Gm* and *fadD2Tn/fadD4::Gm* (Figure 5F). These results indicate that the *P. aeruginosa* FadD4 is involved in the production, accumulation, and degradation of lipid-containing cellular compounds such as biofilm, RHL, PHA, or LPS; consequently, these alterations may render different degrees of bacterial virulence.

### The *fadD4* Mutation Increases Mortality of *C. elegans* Worms

A diverse range of secreted molecules have been associated with the degree of bacterial virulence (Lee and Zhang, 2015; Valentini et al., 2018; Panayidou et al., 2020). A modification of the virulence factors observed in the *fadD* mutants reveals changes in the degree of pathogenicity of the PAO1 strain (Kang et al., 2010; Zarzycki-Siek et al., 2013). Thus, we investigated the effect of *fadD* mutations on the virulence of *P. aeruginosa* PAO1 cultures using the nematode *in vivo* virulence model.

**FIGURE 6:**
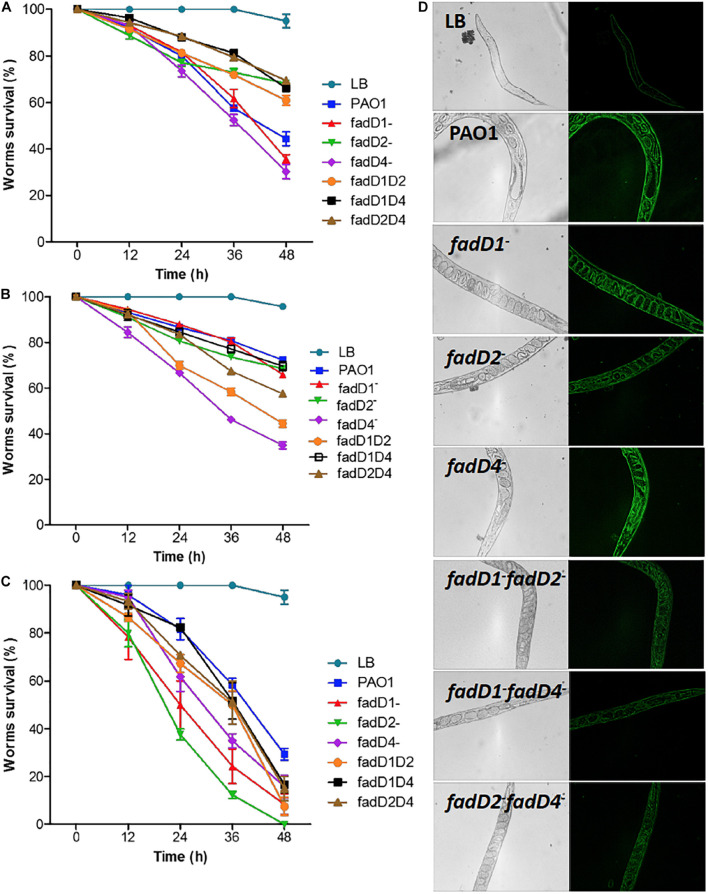
Toxicity of *P. aeruginosa* PAO1 cultures on *C. elegans* worms. **(A,B)** Adult *C. elegans* worms (20 individuals) were incubated with cell-free supernatants obtained after growth of *P. aeruginosa* strains for 24 h **(A)** and 48 h **(B)** or with complete culture media **(C)**. Worm survival was determined at indicated times as described in Section “Materials and Methods.” Bars represent mean ± standard error (SE) of three independent assays. **(D)** Images of oxidative stress responses of CL2166 worms to bacterial strains.

Virulence was determined in *C. elegans* worms using cell-free supernatants of *P. aeruginosa* strains grown in LB medium (slow-killing assay) and live LB bacterial cultures (fast-killing assay). Worms exposed for 48 h to the supernatant from the *fadD4::Gm* single mutant collected from 24-h cultures exhibited survival kinetics similar to that of the *fadD1Tn* mutant and the PAO1 WT strain (Figure 6A), with a nematode survival of ∼30%, compared with ∼45% for the WT strain. In contrast, worm survival increased to ∼60–70% in the case of double mutants *fadD1Tn/fadD2::Gm*, *fadD1Tn/fadD4::Gm*, and *fadD2Tn/fadD4::Gm* and the single mutant *fadD2Tn* (Figure 6A).

Because differential amounts of virulence factors were observed when using 24-h culture supernatants, the supernatants obtained at 48 h were also used to evaluate their effect on worm survival. The worm survival kinetics showed that supernatants from the single mutant *fadD4::Gm* and the double mutants *fadD1Tn/fadD2::Gm* and *fadD2Tn/fadD4::Gm* were more toxic than those from the WT strain and other mutants (Figure 6B). Additionally, the ability to kill *C. elegans* worms was tested with a fast-killing procedure. As shown in Figure 6C, among the *P. aeruginosa* strains tested for slow killing, the mutants *fadD1Tn* (24% survival), *fadD2Tn* (12% survival), and *fadD4::Gm* (35% survival) were the most lethal to adult *C. elegans* worms. Double mutants *fadD1Tn/fadD2::Gm* (50% survival), *fadD1Tn/fadD4::Gm* (52% survival), and *fadD2Tn/fadD4::Gm* (51% survival), as well as WT (58% survival), were moderately less lethal in the first 36 h than others. The *fadD4::Gm* mutant showed a similar increase in lethality in both the slow- and fast-killing assays compared to the WT strain.

The induction of reactive oxygen species (ROS) has been described as the death mechanism associated with virulence factors (Lau et al., 2004); therefore, we used the worms *C. elegans* CL2166 to monitor ROS induction as an effect of the lethality of *P. aeruginosa* cultures. Images show a significant increase in the fluorescence of the worms exposed to 48-h supernatants of cultures of the PAO1 WT and the single mutants *fadD1Tn*, *fadD2Tn*, and *fadD4::Gm*, while the ROS induction was minor in worms exposed to double mutants (Figure 6D). These results indicate that in the *C. elegans* model, ROS generation is not the main mechanism of virulence observed by the single *fadD* mutants of *P. aeruginosa*; however, ROS induction diminished in the double mutants, suggesting a possible implication in cytotoxicity. Thus, our findings confirm that the *fadD1*, *fadD2*, and *fadD4* genes are implicated in the virulence of *P. aeruginosa*, suggesting that this genetic redundancy is a property that confers to this bacterium the ability to respond effectively during infection/pathogenesis processes on hosts or in colonization of ecological niches.

### Correlation Between *fadD* Mutants and Virulence Factors

**FIGURE 7:**
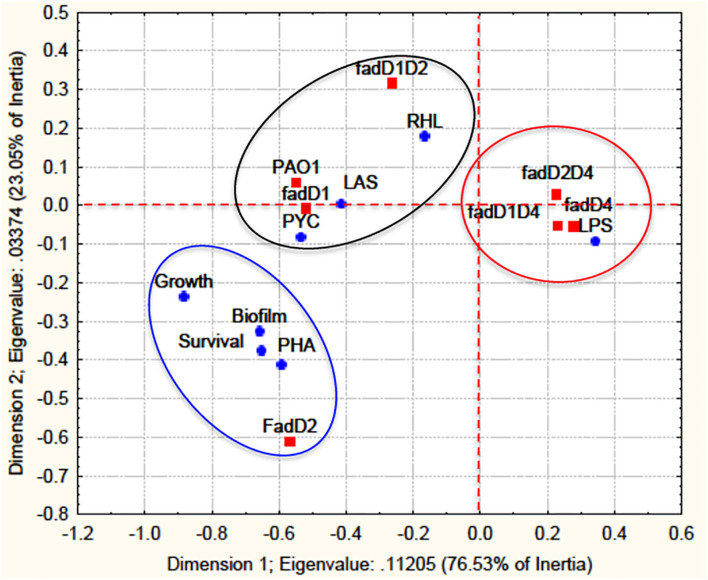
A statistical correlation analysis of virulence factors with *fadD* mutants. A correlation analysis of virulence factor data from the mutagenesis approach was performed by multivariate exploratory techniques, with 2-D PCA correspondence analysis of frequencies with/without grouping variables using the STATISTICA Software System 8.0, Stat Soft. Inc. The *fadD* mutants are designated response variables (independent) and virulence factors as dependent variables. Survival, worm survival; Growth, growth in AT and FA; LAS, QS-*las/rhl* induction; PYC, pyocyanin; RHL, rhamnolipids; PHA, polyhydroxyalkanoates; LPS, lipopolysaccharides; Biofilm, biofilm formation.

Quantitative values of all virulence factors determined in the *fadD* mutants were analyzed using a statistical correlation analysis between genotypes and phenotypes. Based on the correlation analysis, the virulence factors were classified into three groups according to the dependent variables (Figure 7). The *fadD1Tn* and *fadD1Tn/fadD2::Gm* mutants were grouped with the PAO1 WT strain and correlated with the virulence factors AHL-dependent luminescence, rhamnolipids, and pyocyanin (black circle in Figure 7). The second group correlated the single mutant *fadD2Tn* with growth on fatty acids, biofilm production, polyhydroxyalkanoate content, and worm survival (blue circle in Figure 7). Interestingly, the third group correlated the single mutant *fadD4::Gm* and its respective double mutants *fadD1Tn/fadD4::Gm* and *fadD2Tn/fadD4::Gm* with the response factor, LPS content (red circle in Figure 7). This analysis indicates the potential dependence between LPS content and the *fadD4* mutation, suggesting the possible participation of LPS in the increased degree of pathogenicity in the *fadD4* mutant in the worm survival assay.

### The *fadD4* Mutation Modifies Lipopolysaccharides Content and Structure

Lipopolysaccharides are components of the outer membrane of Gram-negative bacteria and are considered to be important virulence factors related to acute *P. aeruginosa* infections and pathogenesis (Lam et al., 2011; Cullen et al., 2015). Modulation of LPS synthesis and structure results in immune evasion, persistent inflammation, and antimicrobial resistance and is important in the adaptation of opportunistic pathogens such as *P. aeruginosa* to chronic infections in the respiratory system (Cullen et al., 2015; Moser et al., 2021). In addition, the LPS structure can be modified at the level of lipid A or antigen O as described for bacteriophage-resistant *P. aeruginosa* PAO1 (Latino et al., 2017).

Thus, we determined the LPS content and characterized the nature of its lipidic component to elucidate FadD4 participation in their synthesis/degradation and correlation with *P. aeruginosa* pathogenicity.

**FIGURE 8:**
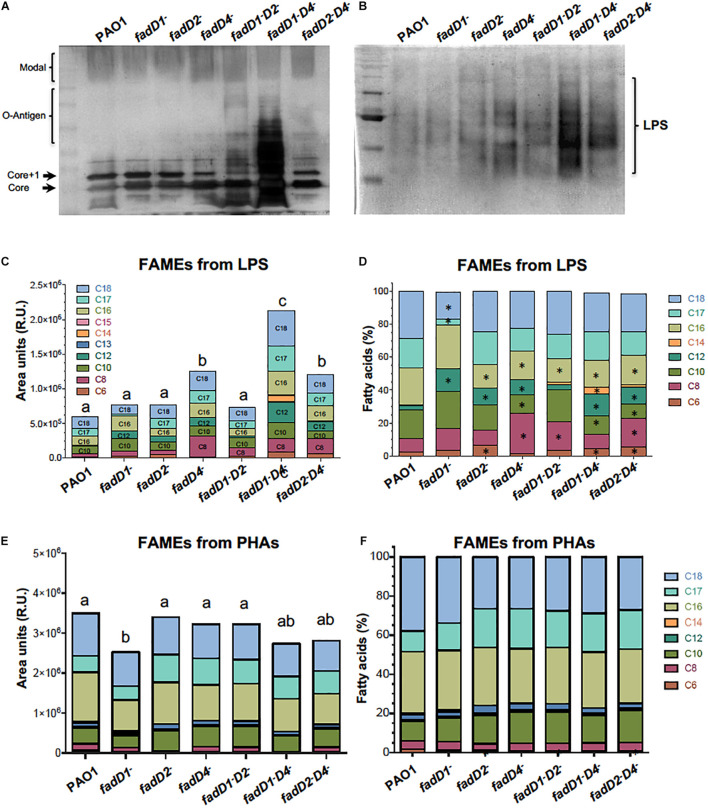
An analysis of LPS and PHA in the *fadD* mutants of *P. aeruginosa* PAO1. Bacterial cultures were grown in Luria broth (LB) medium for 48 h at 37°C. Cells were harvested and LPS isolated and analyzed as described in Section “Materials and Methods.” **(A,B)** Images of silver-stained polyacrylamide gels showing LPS isolated using an extraction method **(A)** (Latino et al., 2017) and methanol–chloroform method **(B)** (Mirzaei et al., 2011). **(C–F)** FA content in LPS and PHAs isolated from cultures of PAO1 strains, determined by fatty acid methyl ester (FAME) derivatization and gas chromatography–mass spectrometry (GC-MS) (Campos-García et al., 2018). **(C,E)** Quantitation of FA of LPS and PHAs isolated from each strain. **(D,F)** Proportion of each FA contained in the LPS and PHAs of respective determinations (%). Chain lengths of FA are indicated as carbon numbers (C_6_–C_18_). Bars represent the mean of three independent experiments. One-way ANOVA with a Bonferroni *post hoc* test was used to compare treatments with respect to control. Significant difference (*P* < 0.05) vs. control is denoted by lowercase letters or asterisks (*).

As described above, the LPS content (determined by measuring disaccharide content) was increased in the *fadD1Tn* and *fadD4::Gm* mutants, as well as in the double mutants *fadD1Tn/fadD4::Gm* and *fadD2Tn/fadD4::Gm* (Figure 5F). An analysis of LPS isolated from bacterial pellets on polyacrylamide gels and silver staining (Latino et al., 2017) revealed a significant increase in the amount of LPS in the double mutant *fadD1Tn/fadD4::Gm* and a minor increase in the *fadD1Tn/fadD2::Gm* and *fadD2Tn/fadD4::Gm* mutants (Figure 8A). In agreement, when the LPS samples were isolated by a different method (Mirzaei et al., 2011) and analyzed on gels under conditions that focused on the LPS-O antigen, gel images showed that the mutant *fadD4::Gm* and the double mutants *fadD1Tn/fadD4::Gm* and *fadD2Tn/fadD4::Gm* contained increased amounts of LPS compared to the WT and other mutants, as well as differential patterns of bands between them (Figure 8B). These findings indicate that in addition to the increased LPS content in the *fadD4::Gm* and *fadD1Tn/fadD4::Gm* mutant, the LPS structure could be modified at the level of lipid A (lipidic component of LPS) or antigen O (carbohydrate component of LPS).

Modifications in the acyl groups of lipid A have been described to occur in LPS of *P. aeruginosa* strains from diverse sources (Lam et al., 2011). To elucidate whether LPS is modified at the level of lipid A structure, the LPS isolated from the *P. aeruginosa* strains were analyzed based on the fatty acid content of lipid A by quantification of derived FAMEs from LPS. The FAME content was significantly increased in the *fadD4::Gm* mutant, as well as its double mutants *fadD1Tn/fadD4::Gm* and *fadD2Tn/fadD4::Gm*, in comparison to the WT strain (Figure 8C), confirming an increase in the LPS content in agreement with LPS quantitation by saccharide determination and gel observation (Figures 5F, 8A,B). Considering the nature of the fatty acids contained in the lipid A of LPS, the analyzed proportion of FAMEs showed that the FAs with chain lengths C_16_, C_12_, C_10_, and C_8_ were modified in the LPS from the *fadD4::Gm* mutant, as well as in the double mutants, compared with the LPS from PAO1 WT (Figures 8C,D). These data confirm that the structure of LPS was modified at the level of the acyl group content in the structure of the lipid A component. Additionally, the quantitation of PHA by FAME determination showed that only the *fadD1* mutant contained decreased amounts of alkanoic acids, and a slight modification of alkanoic acid proportions was observed in the PHA from mutants (Figures 8E,F). Therefore, these results suggest that in *P. aeruginosa* PAO1, the LPS content and, importantly, the acyl groups of FA content in lipid A were modified by the effect of the *fadD4* mutation, which could be associated with the increase in toxicity of the cultures and supernatants observed in the worm survival assays.

### The *fadD4* Mutation Modifies the Pyrogenic Response to Lipopolysaccharides in Rats

Lipopolysaccharides (intraperitoneally administered) is known to be a pyrogenic agent in animals, consisting of *O*-polysaccharide, R-core, and lipid A components, each of which can stimulate the innate immune response. In this sense, structural modifications on lipid A are observed in the number, position, the nature of the linked acyl groups, and on phosphate groups (Pier, 2007; Lam et al., 2011; Moser et al., 2021). Pyrogenic properties associated with the lipid A moiety of LPS are transduced by the TLR4/MD2 receptor of the mammalian innate immune system (Behzadi et al., 2021b). On the other hand, picomolar levels of lipid A induce macrophages to synthesize potent mediators of inflammation, such as TNF-α and IL-1β (Zasłona et al., 2017). In *P. aeruginosa*, the conserved lipid A unit is a *bis*-phosphorylated disaccharide of glucosamine, typically with four to seven acyl chains, and has been also described to consist of four to six FA acyl chains (Simpson and Trent, 2019). Modifications of lipid A from LPS affect several physiological processes in Gram-negative bacteria (e.g., effects on the permeability of the outer membrane, recognition by immune cells, and antimicrobial resistance). Many bacteria utilize lipid A modifications to evade recognition by the mammalian immune response (Simpson and Trent, 2019), and studies have indicated that the number and carbon chain length of acyl groups of FA are critical for TLR4 activation, which can modify the magnitude of the immune response (Harada et al., 1994; McIsaac et al., 2012; Lo Sciuto et al., 2019). Complementary to FA degradation, mutations in enzymes involved in *de novo* FA biosynthesis, such as FabF1/F2 from *Rhizobium leguminosarum*, modify the content and type of FA from the lipid A of LPS and consequently modify adaptability and colonization (Vanderlinde et al., 2009).

**FIGURE 9:**
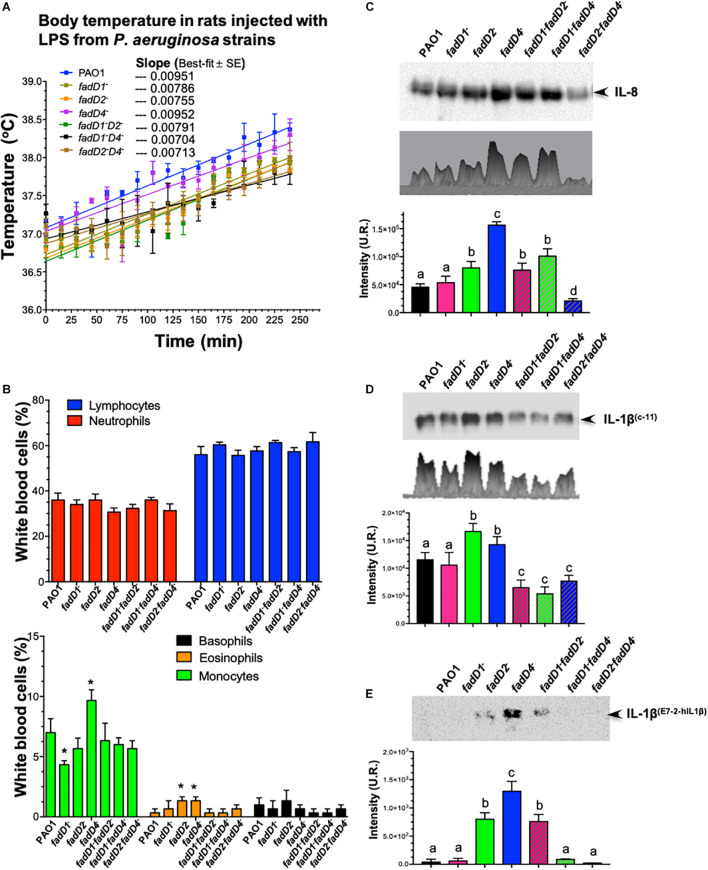
The effect of LPS from *fadD* mutants of *P. aeruginosa* PAO1 on body temperature in rats and immunodetection of interleukins. **(A)** Change in temperature in Wistar rats in response to intraperitoneal injections of LPS isolated from the *P. aeruginosa* PAO1 strains. A dose of 50 μg/kg induced fever in all rats after injection (*n* = 3). Lineal correlation curves are shown, and values of slopes of best fit are indicated. **(B)** Determination of proportions of white blood cells in rats treated with LPS samples. **(C–E)** Immunodetection of interleukins in the serum of rats after LPS treatment: **(C)** IL-8 using anti-IL-8 antibody (sc376750, Santa Cruz Biotechnology), **(D)** IL-1β using anti-IL-1β (C-11) antibody (sc-52012, Santa Cruz Biotechnology), and **(E)** IL-1β using anti-IL-1β (E7-2-hIL1β) antibody (sc-32294, Santa Cruz Biotechnology). Immunodetection of interleukins on western blot membranes was analyzed by densitometry using the Image Lab 6.0.1 software. One-way ANOVA with a Bonferroni *post hoc* test was used to compare treatments with respect to control. Significant difference (*P* < 0.05) vs. control is denoted by lowercase letters or asterisks (*).

To confirm that the mutation in the *fadD4* gene modifies the lipid A structure and, consequently, correlates with the degree of bacterial pathogenicity associated with LPS content and structural composition, a systemic inflammation model in rodents to test the LPS immunogenicity was carried out. The body temperature of the animals administered with LPS at the same concentration increased with time, as can be observed in the WT strain, in which the linear correlation for the PAO1 WT-LPS showed a value of slope = 0.00951 (Figure 9A). A similar increase was observed in the *fadD4::Gm* mutant (slope = 0.00952), while a moderate increase in temperature (lower slope) was observed for the single mutants *fadD1Tn* (slope = 0.00786) and *fadD2Tn* (slope = 0.00755) and the double mutant *fadD1Tn/fadD2::Gm* (slope = 0.00791). Interestingly, animals injected with LPS from the double mutants *fadD1Tn/fadD4::Gm* and *fadD2Tn/fadD4::Gm* showed a minor increase in body temperature, with slopes of 0.00704 and 0.00713, respectively (Figure 9A). In addition, all the rats treated with LPS presented similar symptoms: less active, disinclined to feed, piloerection, hunched posture, ataxic, and weak, thus demonstrating that LPS induced systemic inflammation in animals, with the effect of the *fadD* mutations being associated with this response.

### The *fadD4* Mutation Modifies the Antigenic Response to Lipopolysaccharides in Rats

In the mammalian inflammatory response, interleukin-8 (IL-8) is a chemotactic leukocyte-activating cytokine produced by various types of cells upon stimulation with inflammatory stimuli and exerting a variety of functions on leukocytes of the neutrophil type (Harada et al., 1994).

In our rodent inflammation model, an analysis of leucocyte proportions in the blood of the LPS-treated rats showed that LPS from the *fadD4::Gm* mutant slightly decreased the number of neutrophils, but increased the proportion of monocytes and eosinophils in the bloodstream, compared to the WT strain (Figure 9B). In addition, a western blotting analysis of the blood serum of the LPS-treated rats showed that the LPS from all strains were capable of inducing secretion/production of IL-8 at differential levels, with the single mutants *fadD2* and *fadD4* presenting significantly increased production of IL-8 compared to the LPS from the WT strain (Figure 9C). LPS from the *fadD4* mutant induced the highest level of IL-8. The double mutants *fadD1Tn/fadD2::Gm* and *fadD1Tn/fadD4::Gm* also increased the expression levels of IL-8; however, the double mutant *fadD2Tn/fadD4::Gm* induced lower levels of IL-8 secretion/production than all other strains.

It well known that bacterial LPS also induces the secretion/production of IL-1β in addition to IL-8, which is produced in response to LPS as well as TNF-α (Zasłona et al., 2017). An analysis of IL-1β production in the serum of LPS-treated animals (Figures 9D,E) revealed higher IL-1β levels in the case of *fadD2* and *fadD4* mutants, but interestingly, IL-1β levels were diminished when LPS from double mutants were used (Figure 9D). Interestingly, when another anti-IL-1β antibody was used, the IL-1β immunodetection was mainly observed in the serum of LPS-treated animals from the *fadD4* mutant (Figure 9E). These findings indicate that the LPS from the *fadD* mutants differentially induced the immune response (determined by IL-8 and IL-1β secretion/production), dependent on the TLR4/MD2 response transductor. Our findings indicate that the FadD2 and FadD4 acyl-CoA synthetases modify the induction of innate immune response associated with LPS content and lipid A structural conformation. The different acyl groups of the FA composition of lipid A on these mutants indicate that the antigenicity can be differentially modulated, as observed in the IL-8 versus IL-1β induction (Figures 9C–E), suggesting that the acyl-CoA synthetases FadD2 and FadD4 are mainly involved in LPS synthesis and determine the structural conformation of lipid A, whose antigenicity through the induction of inflammatory mediators IL-8 and IL-1β has been associated with transduction of response by the TLR4/MD2 receptor of the mammalian immune system.

The pathogenesis of *P. aeruginosa* associated to TLR4 sharing ligands with TLR2 recognizes LPS by binding to the lipid A component on the outer membrane of bacteria. In cystic fibrosis patients, *P. aeruginosa* produces several different varieties of lipid A, contributing with the degree of virulence, which is associated to hexa-acylated lipid A, the more potent agonist to TLR4 receptor (McIsaac et al., 2012; Behzadi et al., 2021b). About the mechanism bases of signal transduction, TLR4-MD2 heterodimers are the functional receptors of bacterial LPS. Acyl chains within the lipid A region of LPS interact with distinct regions of two TLR4-MD2 heterodimers, where five of the six acyl chains present in lipid A interact with a hydrophobic pocket present in the MD2 component of a TLR4-MD2 heterodimer. In other cases, LPS structures that contain less than six acyl chains in the lipid A have weakened inflammatory activities by interfering with the TLR4-MD2 heterodimer structuration (McIsaac et al., 2012).

In our study, we present elements that contribute to elucidating the role that can be associated with the *fadD* gene redundancy in *P. aeruginosa* PAO1 and their participation in determining the structure and composition of lipid A. As is well known, FA as substrates differentially induce the expression of *fadD* genes and consequently may modify the LPS antigenicity associated with their content and structure. Findings showed that *fadD2* and *fadD4* modified the LPS content and FA content of lipid A, producing differential modulation of LPS antigenicity. In this context, the LPS from the *fadD1* and *fadD2* mutants were less antigenic and pyrogenic in rats than from the WT strain; however, the LPS from the *fadD4* mutant was more antigenic than that from the WT. In agreement, LPS from the *fadD2* and *fadD4* mutants were better inducers of proinflammatory cytokines, IL-1β and IL-8, than the WT. The differential effects by LPS isolated from the *fadD* mutants could be explained in agreement to the roles that the IL-1 (IL-1α and IL-1β forms) plays in the regulation of immune response and inflammation, acting as an activator of T and B lymphocytes and natural killer cells, which require IL-1β for production of the anti-pathogen IFN-γ. In addition, differential induction of immune response may suggest the participation of different pathways such as the activation of the Nod-like receptor 3 (NLRP3) inflammasome, which is important for the activation of innate immune response to some DAMPs and PAMPs (Behzadi et al., 2021b; Lin et al., 2021).

**FIGURE 10:**
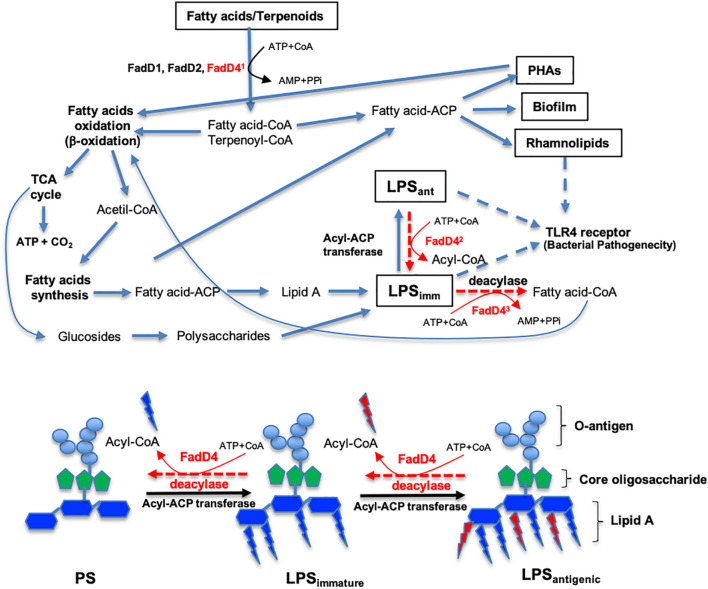
Roles of the acyl-CoA synthetase (FadD4) from *P. aeruginosa* PAO1 on FA catabolism and LPS biosynthesis. The diagram shows the pathways related to fatty acid (FA) and acyclic terpene (AT) catabolism, as well as LPS biosynthesis from FA of external origin or from the FA biosynthesis cycle. Some steps are simplified and can be carried out by several enzymes acting sequentially. The steps where the FadD4 enzyme can act are indicated. First, the activation of FA or AT with coenzyme A (Co-A) directing FA oxidation (β-oxidation) to obtain energy or storage compounds. Second, the elimination/incorporation of some FA on lipid A of the LPS structure to modify antigenicity. Third, a balance between the synthesis and degradation of LPS to recycle its structural compounds. The proposed activity of FadD4 in the modification of the lipid A component of LPS biosynthesis is shown below. PS, polysaccharides without FA lipid A. LPS_imm_ indicates immature lipopolysaccharides or low antigenicity. LPS_ant_ denotes mature lipopolysaccharides or high antigenicity.

Finally, we propose that the pathogenicity of *P. aeruginosa* PAO1 by FadD4 acyl-CoA synthetase involves the bioavailability of substrates such as acyclic terpene and fatty acids as carbon and energy sources by a mechanism of ATP-dependent acyl-CoA activation (Figure 10). This process allows *P. aeruginosa* to produce energy and metabolites *via* FA oxidation (β-oxidation pathway) and tricarboxylic acid cycle. These components can be subsequently utilized for *de novo* FA synthesis to produce storage energy compounds such as polyhydroxyalkanoates and structural components such as rhamnolipids, biofilm, and LPS, where some of the components are composed of acyl groups of FA. Our study demonstrates that FadDs participate in these processes, but more importantly, FadD4 acyl-CoA synthetase, which was involved in the activation of acyl-FA, was directed to LPS biosynthesis (Figure 10). In addition, FadD4 may be suggested to contribute with modification of the FA composition of the lipid A component during LPS biosynthesis, which renders a differential degree of virulence and antigenicity of the *P. aeruginosa* PAO1 LPS. This structural modification associated to FadD4 can also be involved in the elimination or substitution of some acyl-FA on lipid A in the LPS structure, which in turn modifies their antigenicity (Figure 10; see LPS_imm_ and LPS_ant_ types, indicated as LPS with low and strong antigenicity, respectively). Another possibility suggests that FadD4 activity is involved in maintaining the balance between LPS synthesis and degradation, which is important for recycling the lipidic components of LPS. Thus, our study contributes to a deeper understanding of the mechanism involved in the pathogenicity of Gram-negative bacteria, highlighting the importance of lipid A modifications on the LPS virulence factor and the induced immune response in the host by a TLR4-dependent manner.

## Conclusion

Our work confirms that *fadD4* encodes a terpenoyl-CoA synthetase, which is essential for acyclic terpene assimilation and capable of activating fatty acids, similar to the FadD1 and FadD2 homologous enzymes. In addition, FadD4 plays important roles in the synthesis of LPS at the level of the lipid A component, by modifying the acyl groups of FA contained in the lipid A and consequently the amounts of LPS. The structural modification of lipid A was reflected in the degree of virulence of the *P. aeruginosa* PAO1 associated mainly with LPS in a rat model of induction of immune response, which was mediated by the IL-8 and IL-1β interleukins transduced by the TLR4/MD2 receptor.

## Data Availability Statement

The original contributions presented in the study are included in the article/Supplementary Material, further inquiries can be directed to the corresponding author.

## Ethics Statement

The animal study was reviewed and approved by Institutional Committee for Use of Animals of the I.I.Q.B/Universidad Michoacana de San Nicolás de Hidalgo in agreement with NOM-062-ZOO-1999, Ministry of Agriculture of Mexico.

## Author Contributions

JC-G conceptualized and designed the study. LM-A, GO, and AD-P developed the methodology. LM-A and JC-G analyzed and interpreted the data. LM-A, JV, HR-D, EG-P, and JC-G wrote, reviewed, and/or revised the manuscript. JV, HR-D, EG-P, and JC-G provided administrative, technical, or material support. JC-G supervised the study. All authors contributed to the article and approved the submitted version.

## Conflict of Interest

The authors declare that the research was conducted in the absence of any commercial or financial relationships that could be construed as a potential conflict of interest.

## Publisher’s Note

All claims expressed in this article are solely those of the authors and do not necessarily represent those of their affiliated organizations, or those of the publisher, the editors and the reviewers. Any product that may be evaluated in this article, or claim that may be made by its manufacturer, is not guaranteed or endorsed by the publisher.

## Footnotes

1
www.pseudomonas.com
