# Supplementary material for: Participation of Acyl-Coenzyme A Synthetase FadD4 of Pseudomonas aeruginosa PAO1 in Acyclic Terpene/Fatty Acid Assimilation and Virulence by Lipid A Modification
Source: Front Microbiol. 2021 Nov 16;12:785112. doi: 10.3389/fmicb.2021.785112 (PMC8637051; doi:10.3389/fmicb.2021.785112)
Supplement: Supplementary file 1 [file Data_Sheet_1.docx]

Supplementary Material

# Supplementary Figures and Tables

**Table S1.** Alignment analysis of the acyl-CoA synthetases from *Pseudomonas aeruginosa* PAO1 genome using the FadD1, FadD2, Fcs, Acs1, Acs2, Acs3, and Acs4 as protein sequences query. Data were obtained from the database of NCBI using the blasp software.

| **FadD1 (PAO1) vs PAO1 genome** | | | |  |  |  |  |  |  |
| --- | --- | --- | --- | --- | --- | --- | --- | --- | --- |
|  |  |  |  |  |  |  |  |  |  |
| **Seq Query** | **Query Start** | **Query Stop** | **Seq Subject** | **Product Name** | **Subject Start** | **Subject Stop** | **Alignment Length** | **% Identity** | **E-value** |
|  |  |  |  |  |  |  |  |  |  |
| PA3299 | 1 | 562 | PA3299 | long-chain-fatty-acid--CoA ligase | 1 | 562 | 562 | 100.00 | 0 |
| PA3299 | 1 | 553 | PA3300 | long-chain-fatty-acid--CoA ligase | 1 | 560 | 561 | 60.96 | 0 |
| PA3299 | 72 | 557 | PA2557 | probable AMP-binding enzyme | 67 | 555 | 508 | 33.07 | 0 |
| PA3299 | 29 | 558 | PA3860 | probable AMP-binding enzyme | 33 | 574 | 560 | 29.29 | 0 |
| PA3299 | 22 | 550 | PA4228 | pyochelin biosynthesis protein PchD | 32 | 534 | 535 | 25.23 | 9.99967E-42 |
| PA3299 | 49 | 556 | PA2555 | probable AMP-binding enzyme | 57 | 541 | 521 | 25.91 | 5E-34 |
| PA3299 | 27 | 553 | PA4198 | probable AMP-binding enzyme | 21 | 535 | 557 | 25.31 | 4E-33 |
| PA3299 | 223 | 560 | PA3924 | probable medium-chain acyl-CoA ligase | 197 | 553 | 363 | 27.00 | 5E-32 |
| PA3299 | 72 | 552 | PA0996 | PqsA | 50 | 500 | 494 | 24.70 | 2E-27 |
| PA3299 | 75 | 552 | PA2424 | PvdL | 64 | 554 | 532 | 23.68 | 2E-23 |
| PA3299 | 35 | 551 | PA3327 | probable non-ribosomal peptide synthetase | 453 | 939 | 544 | 25.37 | 4E-22 |
| PA3299 | 29 | 551 | PA2424 | PvdL | 1133 | 1622 | 548 | 23.54 | 1E-19 |
| PA3299 | 206 | 551 | PA2399 | pyoverdine synthetase D | 1704 | 2066 | 373 | 26.01 | 7E-19 |
| PA3299 | 206 | 551 | PA2399 | pyoverdine synthetase D | 643 | 1005 | 373 | 26.01 | 2E-18 |
| PA3299 | 41 | 551 | PA2424 | PvdL | 2203 | 2689 | 545 | 24.04 | 9E-18 |
| PA3299 | 35 | 488 | PA1617 | probable AMP-binding enzyme | 22 | 456 | 480 | 24.38 | 9E-18 |
| PA3299 | 203 | 552 | PA4733 | acetyl-coenzyme A synthetase | 249 | 613 | 389 | 23.91 | 2E-17 |
| PA3299 | 75 | 552 | PA3568 | probable acetyl-coa synthetase | 109 | 595 | 515 | 22.91 | 8E-17 |
| PA3299 | 16 | 545 | PA3327 | probable non-ribosomal peptide synthetase | 1467 | 1963 | 556 | 24.10 | 2E-15 |
| PA3299 | 48 | 552 | PA0887 | acetyl-coenzyme A synthetase | 106 | 616 | 530 | 22.26 | 2E-14 |
| PA3299 | 22 | 559 | PA2402 | probable non-ribosomal peptide synthetase | 506 | 1006 | 563 | 22.74 | 3E-13 |
| PA3299 | 358 | 551 | PA2305 | AmbB | 521 | 721 | 203 | 28.57 | 3E-13 |
| PA3299 | 22 | 551 | PA2402 | probable non-ribosomal peptide synthetase | 3033 | 3525 | 552 | 21.74 | 2E-12 |
| PA3299 | 189 | 551 | PA2424 | PvdL | 3843 | 4230 | 404 | 25.00 | 5E-12 |
| PA3299 | 201 | 551 | PA2400 | PvdJ | 1672 | 2038 | 390 | 23.08 | 3E-11 |
| PA3299 | 201 | 551 | PA2402 | probable non-ribosomal peptide synthetase | 4674 | 5040 | 390 | 23.08 | 6E-11 |
| PA3299 | 48 | 487 | PA4226 | dihydroaeruginoic acid synthetase | 578 | 976 | 461 | 23.64 | 8E-10 |
| PA3299 | 199 | 551 | PA1215 | hypothetical protein | 66 | 419 | 376 | 25.27 | 9E-10 |
| PA3299 | 325 | 551 | PA2400 | PvdJ | 734 | 975 | 247 | 24.70 | 3E-09 |
| PA3299 | 206 | 551 | PA1221 | hypothetical protein | 161 | 505 | 364 | 25.27 | 3E-09 |
| PA3299 | 195 | 487 | PA2302 | AmbE | 576 | 873 | 311 | 22.51 | 2E-08 |
| PA3299 | 92 | 551 | PA2402 | probable non-ribosomal peptide synthetase | 2055 | 2479 | 484 | 22.31 | 7E-08 |
| PA3299 | 354 | 558 | PA4078 | probable nonribosomal peptide synthetase | 279 | 498 | 222 | 25.68 | 8E-07 |
|  |  |  |  |  |  |  |  |  |  |
| **FadD2 (PAO1) vs PAO1 genome** | | | |  |  |  |  |  |  |
|  |  |  |  |  |  |  |  |  |  |
| PA3300 | 1 | 562 | PA3300 | long-chain-fatty-acid--CoA ligase | 1 | 562 | 562 | 100.00 | 0 |
| PA3300 | 1 | 560 | PA3299 | long-chain-fatty-acid--CoA ligase | 1 | 553 | 561 | 60.96 | 0 |
| PA3300 | 25 | 562 | PA2557 | probable AMP-binding enzyme | 19 | 550 | 552 | 31.70 | 0 |
| PA3300 | 25 | 559 | PA3860 | probable AMP-binding enzyme | 29 | 568 | 564 | 30.32 | 0 |
| PA3300 | 24 | 557 | PA4228 | pyochelin biosynthesis protein PchD | 34 | 534 | 537 | 25.70 | 2.99878E-43 |
| PA3300 | 30 | 562 | PA4198 | probable AMP-binding enzyme | 24 | 537 | 563 | 26.82 | 8E-36 |
| PA3300 | 223 | 562 | PA3924 | probable medium-chain acyl-CoA ligase | 197 | 547 | 360 | 30.00 | 7E-35 |
| PA3300 | 48 | 559 | PA2555 | probable AMP-binding enzyme | 56 | 537 | 524 | 24.81 | 4E-32 |
| PA3300 | 212 | 562 | PA0996 | PqsA | 161 | 503 | 358 | 27.37 | 3E-29 |
| PA3300 | 28 | 560 | PA2424 | PvdL | 1132 | 1624 | 548 | 24.45 | 3E-26 |
| PA3300 | 47 | 558 | PA3327 | probable non-ribosomal peptide synthetase | 469 | 939 | 521 | 26.10 | 1E-25 |
| PA3300 | 14 | 559 | PA2424 | PvdL | 7 | 554 | 606 | 26.07 | 1E-24 |
| PA3300 | 22 | 562 | PA4733 | acetyl-coenzyme A synthetase | 77 | 616 | 573 | 24.43 | 5E-25 |
| PA3300 | 50 | 562 | PA0887 | acetyl-coenzyme A synthetase | 108 | 619 | 555 | 24.50 | 3E-24 |
| PA3300 | 54 | 562 | PA3568 | probable acetyl-coa synthetase | 93 | 598 | 532 | 25.94 | 1E-23 |
| PA3300 | 19 | 558 | PA2402 | probable non-ribosomal peptide synthetase | 506 | 998 | 553 | 23.87 | 5E-20 |
| PA3300 | 19 | 558 | PA2402 | probable non-ribosomal peptide synthetase | 3033 | 3525 | 554 | 24.01 | 9E-20 |
| PA3300 | 207 | 558 | PA2400 | PvdJ | 1678 | 2038 | 381 | 25.20 | 3E-19 |
| PA3300 | 26 | 552 | PA3327 | probable non-ribosomal peptide synthetase | 1477 | 1963 | 554 | 22.92 | 3E-19 |
| PA3300 | 207 | 558 | PA2402 | probable non-ribosomal peptide synthetase | 4680 | 5040 | 381 | 25.20 | 9E-19 |
| PA3300 | 41 | 558 | PA2424 | PvdL | 2203 | 2689 | 541 | 24.58 | 1E-18 |
| PA3300 | 50 | 496 | PA1617 | probable AMP-binding enzyme | 37 | 456 | 472 | 25.64 | 1E-18 |
| PA3300 | 28 | 558 | PA2305 | AmbB | 243 | 721 | 540 | 23.70 | 1E-17 |
| PA3300 | 207 | 558 | PA2399 | pyoverdine synthetase D | 1705 | 2066 | 385 | 25.71 | 3E-17 |
| PA3300 | 47 | 558 | PA2400 | PvdJ | 498 | 975 | 538 | 22.68 | 3E-17 |
| PA3300 | 207 | 558 | PA2399 | pyoverdine synthetase D | 644 | 1005 | 385 | 25.71 | 7E-17 |
| PA3300 | 365 | 558 | PA1215 | hypothetical protein | 228 | 419 | 196 | 32.14 | 1E-16 |
| PA3300 | 354 | 561 | PA2402 | probable non-ribosomal peptide synthetase | 2265 | 2482 | 223 | 29.15 | 2E-13 |
| PA3300 | 205 | 493 | PA2302 | AmbE | 586 | 871 | 304 | 24.67 | 2E-13 |
| PA3300 | 191 | 558 | PA2424 | PvdL | 3845 | 4230 | 401 | 22.69 | 4E-13 |
| PA3300 | 200 | 559 | PA4078 | probable nonribosomal peptide synthetase | 135 | 489 | 372 | 23.12 | 7E-13 |
| PA3300 | 207 | 515 | PA4225 | pyochelin synthetase | 652 | 959 | 321 | 23.36 | 1E-11 |
| PA3300 | 50 | 561 | PA1997 | probable AMP-binding enzyme | 115 | 622 | 547 | 23.03 | 5E-11 |
| PA3300 | 182 | 495 | PA4226 | dihydroaeruginoic acid synthetase | 662 | 976 | 333 | 25.23 | 7E-10 |
| PA3300 | 207 | 558 | PA1221 | hypothetical protein | 162 | 505 | 370 | 24.86 | 1E-09 |
| PA3300 | 185 | 562 | PA2893 | putative very-long chain acyl-CoA synthetase | 184 | 570 | 408 | 23.53 | 2E-09 |
|  |  |  |  |  |  |  |  |  |  |
|  |  |  |  |  |  |  |  |  |  |
| **Fcs *(P. Fluorescens)* vs PAO1 genome** | | | | |  |  |  |  |  |
|  |  |  |  |  |  |  |  |  |  |
| unnamed | 25 | 459 | PA1617 | probable AMP-binding enzyme | 19 | 442 | 456 | 25.66 | 3E-18 |
| unnamed | 76 | 419 | PA3299 | long-chain-fatty-acid--CoA ligase | 84 | 439 | 373 | 23.86 | 2E-14 |
| unnamed | 179 | 419 | PA3300 | long-chain-fatty-acid--CoA ligase | 204 | 447 | 257 | 24.51 | 1E-13 |
| unnamed | 162 | 419 | PA0996 | PqsA | 135 | 382 | 279 | 27.60 | 0.000000004 |
| unnamed | 44 | 425 | PA2557 | probable AMP-binding enzyme | 47 | 440 | 413 | 24.46 | 0.000000007 |
| unnamed | 43 | 482 | PA3327 | probable non-ribosomal peptide synthetase | 472 | 885 | 456 | 22.37 | 0.00000001 |
| unnamed | 10 | 451 | PA2424 | PvdL | 7 | 455 | 501 | 24.75 | 0.0000002 |
| unnamed | 39 | 488 | PA4228 | pyochelin biosynthesis protein PchD | 57 | 489 | 478 | 25.94 | 0.0000001 |
| unnamed | 44 | 211 | PA2424 | PvdL | 3740 | 3891 | 171 | 25.73 | 0.0000003 |
|  |  |  |  |  |  |  |  |  |  |
| **Acs1 (*M. Hydrocarbonoclasticus)* vs PAO1 genome** | | | | |  |  |  |  |  |
|  |  |  |  |  |  |  |  |  |  |
| unnamed | 10 | 536 | PA3924 | probable medium-chain acyl-CoA ligase | 18 | 548 | 539 | 38.03 | 0 |
| unnamed | 39 | 531 | PA4198 | probable AMP-binding enzyme | 43 | 534 | 501 | 35.13 | 0 |
| unnamed | 11 | 531 | PA2557 | probable AMP-binding enzyme | 19 | 547 | 553 | 26.40 | 8.00001E-41 |
| unnamed | 4 | 531 | PA3860 | probable AMP-binding enzyme | 22 | 568 | 571 | 27.50 | 6E-39 |
| unnamed | 42 | 531 | PA3300 | long-chain-fatty-acid--CoA ligase | 52 | 559 | 524 | 26.34 | 1E-38 |
| unnamed | 59 | 533 | PA3299 | long-chain-fatty-acid--CoA ligase | 63 | 554 | 506 | 24.90 | 2E-35 |
| unnamed | 39 | 528 | PA4228 | pyochelin biosynthesis protein PchD | 59 | 533 | 510 | 23.53 | 9E-25 |
| unnamed | 42 | 530 | PA3327 | probable non-ribosomal peptide synthetase | 474 | 939 | 509 | 25.34 | 6E-24 |
| unnamed | 165 | 531 | PA0996 | PqsA | 141 | 500 | 384 | 26.82 | 4E-23 |
| unnamed | 39 | 530 | PA2402 | probable non-ribosomal peptide synthetase | 536 | 998 | 508 | 25.00 | 1E-20 |
| unnamed | 42 | 466 | PA1617 | probable AMP-binding enzyme | 39 | 455 | 456 | 24.12 | 5E-20 |
| unnamed | 39 | 530 | PA2402 | probable non-ribosomal peptide synthetase | 3063 | 3525 | 509 | 24.75 | 2E-19 |
| unnamed | 7 | 532 | PA4733 | acetyl-coenzyme A synthetase | 73 | 614 | 586 | 22.70 | 2E-19 |
| unnamed | 35 | 532 | PA3568 | probable acetyl-coa synthetase | 80 | 596 | 547 | 23.58 | 2E-19 |
| unnamed | 7 | 532 | PA0887 | acetyl-coenzyme A synthetase | 74 | 617 | 572 | 20.45 | 1E-16 |
| unnamed | 26 | 466 | PA2424 | PvdL | 1142 | 1556 | 461 | 26.25 | 4E-15 |
| unnamed | 42 | 530 | PA2402 | probable non-ribosomal peptide synthetase | 2016 | 2479 | 508 | 24.21 | 2E-14 |
| unnamed | 32 | 533 | PA2555 | probable AMP-binding enzyme | 50 | 539 | 521 | 21.50 | 7E-12 |
| unnamed | 172 | 530 | PA2399 | pyoverdine synthetase D | 1700 | 2066 | 389 | 22.88 | 3E-11 |
| unnamed | 182 | 530 | PA2400 | PvdJ | 623 | 975 | 371 | 25.88 | 2E-11 |
| unnamed | 172 | 530 | PA2399 | pyoverdine synthetase D | 639 | 1005 | 379 | 22.43 | 3E-11 |
| unnamed | 41 | 471 | PA2302 | AmbE | 475 | 878 | 446 | 24.89 | 4E-11 |
| unnamed | 172 | 536 | PA4078 | probable nonribosomal peptide synthetase | 136 | 494 | 385 | 23.64 | 1E-10 |
| unnamed | 184 | 466 | PA4225 | pyochelin synthetase | 659 | 940 | 295 | 26.10 | 2E-10 |
| unnamed | 38 | 465 | PA2424 | PvdL | 3737 | 4152 | 448 | 22.99 | 8E-10 |
| unnamed | 173 | 530 | PA2305 | AmbB | 376 | 721 | 377 | 24.14 | 2E-09 |
| unnamed | 162 | 533 | PA2893 | putative very-long chain acyl-CoA synthetase | 192 | 569 | 399 | 22.06 | 2E-08 |
| unnamed | 184 | 524 | PA1215 | hypothetical protein | 85 | 413 | 348 | 20.69 | 4E-08 |
| unnamed | 182 | 509 | PA2424 | PvdL | 2336 | 2663 | 344 | 24.42 | 6E-08 |
| unnamed | 49 | 532 | PA3327 | probable non-ribosomal peptide synthetase | 1510 | 1971 | 507 | 21.89 | 6E-06 |
| unnamed | 129 | 463 | PA4226 | dihydroaeruginoic acid synthetase | 642 | 972 | 348 | 23.56 | 6E-05 |
|  |  |  |  |  |  |  |  |  |  |
|  |  |  |  |  |  |  |  |  |  |
| **Acs2 (*M. Hydrocarbonoclasticus)* vs PAO1 genome** | | | | |  |  |  |  |  |
| unnamed | 3 | 554 | PA3299 | long-chain-fatty-acid--CoA ligase | 2 | 554 | 554 | 63.36 | 0 |
| unnamed | 5 | 555 | PA3300 | long-chain-fatty-acid--CoA ligase | 4 | 562 | 559 | 59.03 | 0 |
| unnamed | 29 | 553 | PA2557 | probable AMP-binding enzyme | 22 | 548 | 547 | 29.62 | 0 |
| unnamed | 69 | 555 | PA3860 | probable AMP-binding enzyme | 79 | 571 | 510 | 30.98 | 0 |
| unnamed | 51 | 550 | PA4228 | pyochelin biosynthesis protein PchD | 60 | 534 | 508 | 27.17 | 2.8026E-45 |
| unnamed | 49 | 554 | PA2555 | probable AMP-binding enzyme | 56 | 539 | 521 | 27.26 | 3.99931E-42 |
| unnamed | 213 | 557 | PA4198 | probable AMP-binding enzyme | 186 | 539 | 369 | 30.89 | 4E-34 |
| unnamed | 48 | 552 | PA2424 | PvdL | 38 | 554 | 568 | 27.11 | 2E-29 |
| unnamed | 228 | 554 | PA3924 | probable medium-chain acyl-CoA ligase | 211 | 545 | 349 | 27.51 | 3E-29 |
| unnamed | 36 | 551 | PA3327 | probable non-ribosomal peptide synthetase | 453 | 939 | 543 | 25.05 | 3E-25 |
| unnamed | 29 | 551 | PA2424 | PvdL | 1132 | 1622 | 542 | 25.46 | 6E-25 |
| unnamed | 73 | 556 | PA0996 | PqsA | 50 | 504 | 496 | 23.59 | 1E-24 |
| unnamed | 16 | 555 | PA3327 | probable non-ribosomal peptide synthetase | 1463 | 1967 | 565 | 24.07 | 1E-22 |
| unnamed | 209 | 551 | PA2424 | PvdL | 3863 | 4230 | 379 | 27.97 | 3E-22 |
| unnamed | 51 | 558 | PA0887 | acetyl-coenzyme A synthetase | 108 | 622 | 536 | 24.63 | 1E-22 |
| unnamed | 39 | 552 | PA4733 | acetyl-coenzyme A synthetase | 98 | 613 | 536 | 24.25 | 2E-21 |
| unnamed | 208 | 551 | PA2402 | probable non-ribosomal peptide synthetase | 4680 | 5040 | 373 | 26.54 | 4E-21 |
| unnamed | 51 | 489 | PA1617 | probable AMP-binding enzyme | 37 | 456 | 467 | 25.27 | 3E-21 |
| unnamed | 208 | 551 | PA2400 | PvdJ | 1678 | 2038 | 373 | 26.54 | 8E-21 |
| unnamed | 76 | 558 | PA3568 | probable acetyl-coa synthetase | 109 | 601 | 512 | 23.63 | 5E-21 |
| unnamed | 30 | 551 | PA2424 | PvdL | 2191 | 2689 | 552 | 23.55 | 2E-20 |
| unnamed | 208 | 551 | PA2399 | pyoverdine synthetase D | 1705 | 2066 | 371 | 27.49 | 2E-20 |
| unnamed | 208 | 551 | PA2399 | pyoverdine synthetase D | 644 | 1005 | 372 | 27.69 | 2E-20 |
| unnamed | 30 | 557 | PA2402 | probable non-ribosomal peptide synthetase | 3043 | 3532 | 546 | 24.18 | 5E-20 |
| unnamed | 30 | 551 | PA2402 | probable non-ribosomal peptide synthetase | 516 | 998 | 540 | 24.26 | 5E-20 |
| unnamed | 30 | 551 | PA2305 | AmbB | 244 | 721 | 536 | 23.69 | 1E-16 |
| unnamed | 43 | 551 | PA2400 | PvdJ | 493 | 975 | 538 | 23.23 | 4E-15 |
| unnamed | 215 | 552 | PA4078 | probable nonribosomal peptide synthetase | 148 | 489 | 362 | 25.14 | 3E-14 |
| unnamed | 403 | 551 | PA2402 | probable non-ribosomal peptide synthetase | 2322 | 2479 | 160 | 32.50 | 4E-13 |
| unnamed | 73 | 497 | PA2302 | AmbE | 495 | 881 | 445 | 22.47 | 2E-12 |
| unnamed | 331 | 551 | PA1215 | hypothetical protein | 201 | 419 | 226 | 26.99 | 2E-10 |
| unnamed | 208 | 488 | PA4225 | pyochelin synthetase | 652 | 940 | 298 | 24.50 | 2E-09 |
| unnamed | 178 | 486 | PA1221 | hypothetical protein | 135 | 446 | 337 | 25.22 | 2E-09 |
| unnamed | 32 | 553 | PA1997 | probable AMP-binding enzyme | 91 | 621 | 561 | 22.46 | 5E-09 |
| unnamed | 69 | 509 | PA4226 | dihydroaeruginoic acid synthetase | 597 | 998 | 458 | 21.40 | 2E-08 |
|  |  |  |  |  |  |  |  |  |  |
|  |  |  |  |  |  |  |  |  |  |
|  |  |  |  |  |  |  |  |  |  |
| **Acs3 (*M. Hydrocarbonoclasticus)* vs PAO1 genome** | | | | |  |  |  |  |  |
| unnamed | 1 | 555 | PA1617 | probable AMP-binding enzyme | 1 | 554 | 556 | 55.40 | 0 |
| unnamed | 35 | 456 | PA2557 | probable AMP-binding enzyme | 44 | 483 | 466 | 24.89 | 1E-27 |
| unnamed | 162 | 446 | PA0996 | PqsA | 152 | 421 | 307 | 28.01 | 4E-25 |
| unnamed | 129 | 536 | PA3860 | probable AMP-binding enzyme | 179 | 578 | 445 | 26.29 | 3E-24 |
| unnamed | 166 | 445 | PA3299 | long-chain-fatty-acid--CoA ligase | 207 | 477 | 297 | 25.59 | 3E-20 |
| unnamed | 166 | 456 | PA3300 | long-chain-fatty-acid--CoA ligase | 207 | 496 | 316 | 26.27 | 4E-20 |
| unnamed | 35 | 510 | PA3924 | probable medium-chain acyl-CoA ligase | 42 | 527 | 517 | 22.24 | 2E-16 |
| unnamed | 38 | 450 | PA4228 | pyochelin biosynthesis protein PchD | 61 | 466 | 443 | 22.57 | 1E-15 |
| unnamed | 141 | 471 | PA2424 | PvdL | 153 | 492 | 376 | 26.33 | 7E-13 |
| unnamed | 36 | 493 | PA3568 | probable acetyl-coa synthetase | 84 | 566 | 511 | 20.94 | 9E-13 |
| unnamed | 46 | 444 | PA3327 | probable non-ribosomal peptide synthetase | 1510 | 1891 | 427 | 24.82 | 3E-12 |
| unnamed | 171 | 493 | PA0887 | acetyl-coenzyme A synthetase | 261 | 587 | 352 | 21.88 | 3E-11 |
| unnamed | 31 | 489 | PA2400 | PvdJ | 497 | 947 | 496 | 24.60 | 5E-11 |
| unnamed | 171 | 454 | PA1215 | hypothetical protein | 83 | 356 | 298 | 26.17 | 2E-10 |
| unnamed | 31 | 474 | PA2555 | probable AMP-binding enzyme | 52 | 492 | 476 | 23.32 | 3E-10 |
| unnamed | 164 | 463 | PA4733 | acetyl-coenzyme A synthetase | 251 | 549 | 324 | 21.60 | 7E-10 |
| unnamed | 166 | 491 | PA4225 | pyochelin synthetase | 652 | 983 | 359 | 24.79 | 2E-09 |
| unnamed | 153 | 455 | PA4226 | dihydroaeruginoic acid synthetase | 676 | 975 | 332 | 24.70 | 2E-08 |
| unnamed | 80 | 446 | PA2402 | probable non-ribosomal peptide synthetase | 2057 | 2405 | 397 | 23.17 | 2E-07 |
| unnamed | 46 | 204 | PA2424 | PvdL | 2221 | 2369 | 161 | 28.57 | 7E-07 |
| unnamed | 166 | 449 | PA1221 | hypothetical protein | 162 | 442 | 316 | 24.68 | 5E-07 |
| unnamed | 38 | 491 | PA2400 | PvdJ | 1562 | 2008 | 490 | 22.86 | 6E-06 |
|  |  |  |  |  |  |  |  |  |  |
|  |  |  |  |  |  |  |  |  |  |
|  |  |  |  |  |  |  |  |  |  |
| **Acs4 (*M. Hydrocarbonoclasticus)* vs PAO1 genome** | | | | |  |  |  |  |  |
| unnamed | 11 | 558 | PA1617 | probable AMP-binding enzyme | 9 | 553 | 551 | 49.55 | 0 |
| unnamed | 169 | 499 | PA0996 | PqsA | 154 | 473 | 366 | 29.23 | 1E-24 |
| unnamed | 132 | 459 | PA3299 | long-chain-fatty-acid--CoA ligase | 171 | 486 | 344 | 27.03 | 2E-22 |
| unnamed | 39 | 460 | PA3860 | probable AMP-binding enzyme | 62 | 503 | 476 | 24.58 | 1E-21 |
| unnamed | 40 | 460 | PA2557 | probable AMP-binding enzyme | 47 | 483 | 466 | 25.11 | 1E-20 |
| unnamed | 171 | 478 | PA3300 | long-chain-fatty-acid--CoA ligase | 207 | 514 | 333 | 29.73 | 8E-20 |
| unnamed | 41 | 499 | PA4198 | probable AMP-binding enzyme | 46 | 508 | 520 | 23.08 | 5E-14 |
| unnamed | 46 | 465 | PA3924 | probable medium-chain acyl-CoA ligase | 51 | 479 | 466 | 22.53 | 1E-10 |
| unnamed | 170 | 537 | PA2424 | PvdL | 166 | 552 | 420 | 23.81 | 2E-10 |
| unnamed | 37 | 207 | PA2399 | pyoverdine synthetase D | 526 | 680 | 180 | 28.33 | 2E-08 |
| unnamed | 37 | 207 | PA2399 | pyoverdine synthetase D | 1587 | 1741 | 180 | 28.33 | 2E-08 |
| unnamed | 37 | 222 | PA2402 | probable non-ribosomal peptide synthetase | 3062 | 3231 | 186 | 24.73 | 4E-08 |
| unnamed | 37 | 222 | PA2402 | probable non-ribosomal peptide synthetase | 535 | 704 | 186 | 24.73 | 4E-08 |
| unnamed | 33 | 465 | PA2555 | probable AMP-binding enzyme | 52 | 475 | 465 | 23.01 | 3E-08 |
| unnamed | 39 | 222 | PA2400 | PvdJ | 501 | 669 | 188 | 27.13 | 6E-08 |
| unnamed | 39 | 222 | PA2424 | PvdL | 1154 | 1321 | 187 | 25.67 | 9E-08 |
| unnamed | 332 | 452 | PA4228 | pyochelin biosynthesis protein PchD | 332 | 464 | 134 | 32.09 | 2E-07 |
| unnamed | 176 | 427 | PA1215 | hypothetical protein | 83 | 325 | 271 | 23.62 | 2E-07 |
| unnamed | 39 | 221 | PA3327 | probable non-ribosomal peptide synthetase | 472 | 636 | 183 | 23.50 | 5E-07 |
| unnamed | 37 | 192 | PA2424 | PvdL | 2210 | 2352 | 156 | 26.92 | 2E-06 |
| unnamed | 37 | 202 | PA2400 | PvdJ | 1559 | 1711 | 169 | 29.59 | 2E-06 |
| unnamed | 37 | 202 | PA2402 | probable non-ribosomal peptide synthetase | 4561 | 4713 | 169 | 29.59 | 5E-06 |
| unnamed | 171 | 432 | PA4225 | pyochelin synthetase | 652 | 913 | 280 | 23.57 | 4E-06 |
| unnamed | 40 | 222 | PA3327 | probable non-ribosomal peptide synthetase | 1502 | 1663 | 191 | 26.70 | 1E-05 |
| unnamed | 14 | 217 | PA4226 | dihydroaeruginoic acid synthetase | 560 | 735 | 204 | 27.45 | 1E-05 |
| unnamed | 37 | 218 | PA2402 | probable non-ribosomal peptide synthetase | 2012 | 2177 | 188 | 26.06 | 1E-04 |


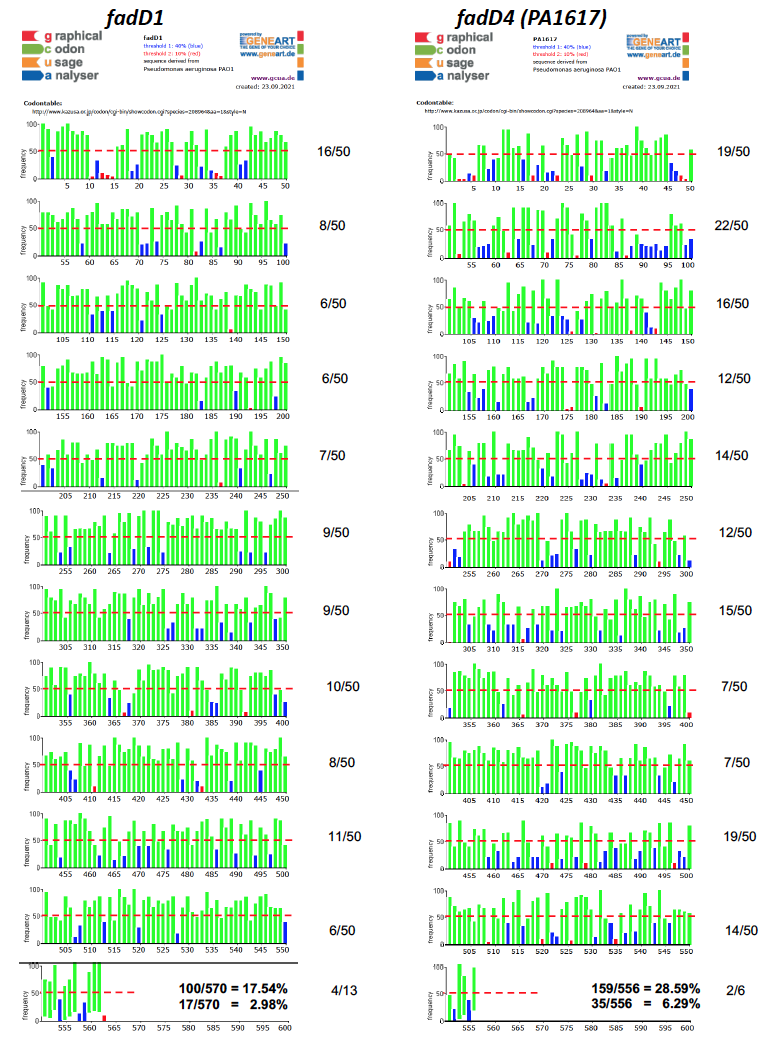


**Figure S1.- Codon usage of *fadD1* and *fadD4* genes.** (Software utilized, Graphical Codon Usage Analyser; Fuhrmann M, Hausherr A, Ferbitz L, Schödl T, Heitzer M, Hegemann P. Plant Mol Biol. 2004 Aug;55(6):869-81. http://dx.doi.org/10.1007/s11103-005-2150-1).
